# Supplementary material for: Loneliness, social isolation, and effects on cognitive decline in patients with dementia: A retrospective cohort study using natural language processing
Source: Alzheimers Dement (Amst). 2025 Jul 28;17(3):e70149. doi: 10.1002/dad2.70149 (PMC12301694; doi:10.1002/dad2.70149)
Supplement: Supplementary file 2 — Supporting Information [file DAD2-17-e70149-s002.docx]

Main analysis with code for Integrating Natural Language Processing to Assess Effect of Loneliness and Social isolation on Cognitive Decline in Dementia: A Retrospective Cohort Analysis

James Myers, MSc ^1,2,9,10^, Tom Stafford, PhD^1,2^, Ivan Koychev, PhD^3,9^, Robert Perneczky, PhD ^2,4,5,6,7,8^, Oliver Bandmann, PhD^2,4^, Nemanja Vaci, PhD^1,2,9,10^*

1. School of Psychology, University of Sheffield, ICOSS, 219 Portobello, Sheffield S1 4DP, United Kingdom
2. Division of Neuroscience, University of Sheffield, Western Bank, Sheffield S10 2TN, United Kingdom
3. Department of Psychiatry, University of Oxford, Warneford Hospital, Warneford Lane, Headington, Oxford OX3 7JX, United Kingdom
4. School of Medicine and Population Health, University of Sheffield, Beech Hill Road, Sheffield S10 2RX, United Kingdom
5. Department of Psychiatry and Psychotherapy, LMU Hospital, Nußbaumstraße 7, 80336 Munich, Germany
6. German Center for Neurodegenerative Diseases (DZNE), Feodor-Lynen-Straße 17, 81377 Munich, Germany
7. Munich Cluster for Systems Neurology (SyNergy), Feodor-Lynen-Straße 17, 81377 Munich, Germany
8. Ageing Epidemiology Research Unit (AGE), School of Public Health, Imperial College London, 90 Wood Lane, London W12 0BZ, United Kingdom
9. Oxford Health NHS Foundation Trust, Warneford Hospital, Warneford Lane, Headington, Oxford OX3 7JX, United Kingdom
10. Rotherham, Doncaster and South Humber NHS Foundation Trust, Woodfield House, Tickhill Road Site, Weston Road, Balby, Doncaster, DN4 8QN, UK

| **Table of Contents** | Page |
| --- | --- |
| **MoCA analysis** | 3 |
| GAMM models | 4 |
| LMER models | 9 |
| MoCA change after the first report | 19 |

MoCA analysis

Raw MoCA observations across the disease duration:

moca2 = subset(moca, diag_dur_y >= -5 & diag_dur_y<=5 & (CompNew == 'Isolation' | CompNew == 'Loneliness' | CompNew=='NoComplaints'))

par(cex=1.1, font.lab=2, font.axis=1.5, bty='n')
plot(moca2$diag_dur_y, moca2$scal_score, xlab='Diagnosis duration (0 - time of diagnosis)',ylab='Cognitive functionality (MoCA)')


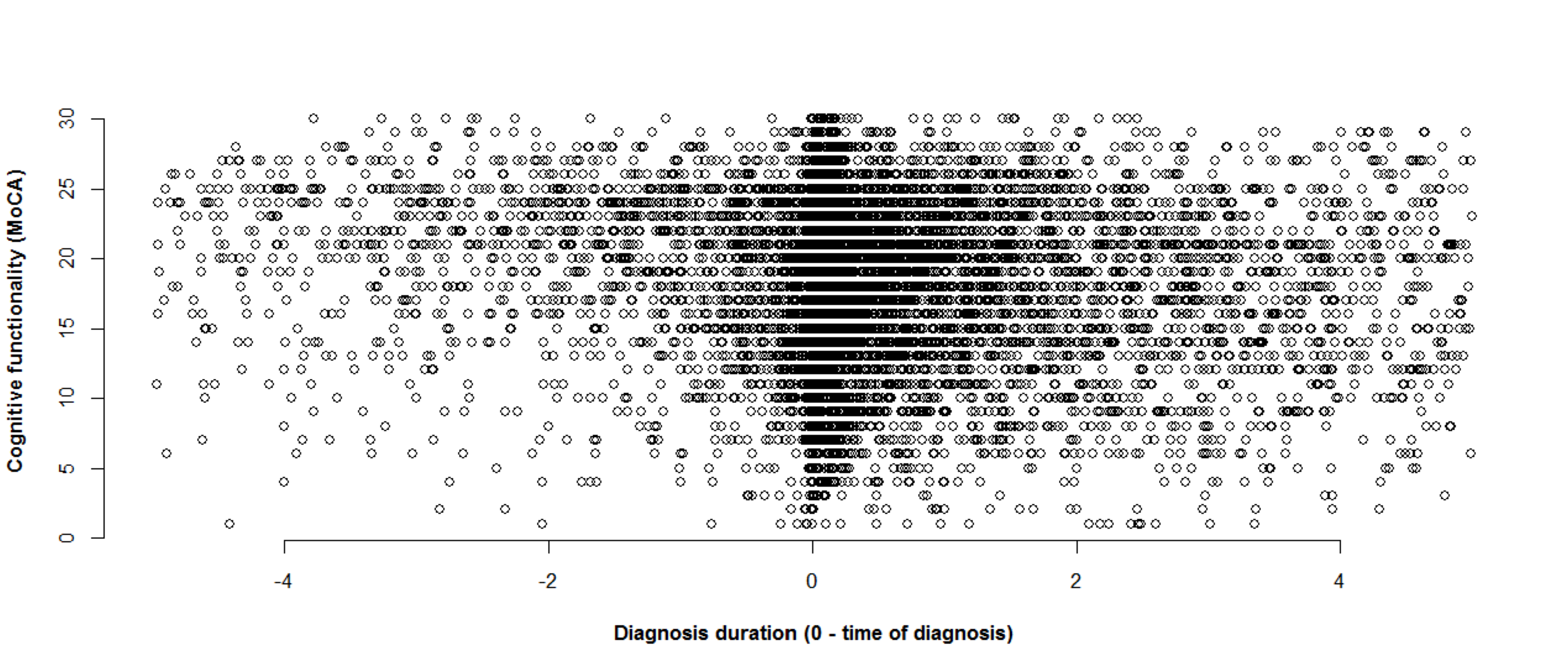


Descriptive Statistics

We then calculated **descriptive statistics** for all main variables across three key groups:

- **No reports** of social isolation or loneliness
- **Reports of social isolation**
- **Reports of loneliness**

For each group, descriptive statistics were calculated for the following variables:

- **Cognitive scores**: MMSE and MoCA (mean, standard deviation)
- **Demographic variables**: Age, sex, marital status, accommodation status
- **Clinical factors**: Dementia diagnosis, depression (based on ICD-10 codes)

These descriptive statistics provided an initial view of how patients differed across the three groups, shedding light on how social isolation and loneliness might intersect with cognitive decline and demographic or clinical factors.

**Summary of Analytical Approach**

By combining social and cognitive measures, along with demographic and clinical data, we aimed to explore the relationship between **social isolation, loneliness, and cognitive function** in dementia patients. This approach allowed us to account for potential confounders such as age, sex, marital status, and depression, while focusing on a critical time period around dementia diagnosis. The descriptive statistics served as the foundation for further inferential analyses, such as examining whether there are significant differences in cognitive decline between patients with and without reports of social isolation or loneliness.

Descriptive statistics

moca <- read.csv('MOCACombinedComplaints.csv')
ICD10 <- read_xlsx('F:/22-ExtendedNewMind/New Mind 2/Data_extract_22072024/icd10_depression.xlsx')
ICD10 = ICD10[,c(1,4)]
names(ICD10)[1]='BRC_ID'

ICD10=ICD10[!duplicated(ICD10$BRC_ID),]

moca = merge(moca, ICD10, by='BRC_ID', all.x=T)

moca2 = subset(moca, diag_dur_y >= -5 & diag_dur_y<=5 & (CompNew == 'Isolation' | CompNew == 'Loneliness' | CompNew=='NoComplaints'))

moca2$ethnicity=as.factor(moca2$ethnicity)
levels(moca2$ethnicity)=c('Other','Other','Other','Other','White')

moca2$marital_status=as.factor(moca2$marital_status)
levels(moca2$marital_status)=c('Partnership','Divorced','Partnership','Divorced','Single','Widowed')

moca2$diag_cause=as.factor(moca2$diag_cause)
levels(moca2$diag_cause)=c('AD','DLB','Other','Other','Other','Other','Unspecified','VaD')

moca2$acco_status=as.factor(moca2$acco_status)
levels(moca2$acco_status)=c('Other','Other','Mainstream Housing','Other','Supported accommodation')

moca2$diagnosis_code=ifelse(is.na(moca2$diagnosis_code),'No',moca2$diagnosis_code)
moca2$diagnosis_code=as.factor(moca2$diagnosis_code)
levels(moca2$diagnosis_code)=c('F32.0','F32.1','F32.2','F32.3','F33.0>','F33.0>','F33.0>','F33.0>','F33.0>','No')

GAMM models

In the first step of the analysis, the data were analyzed using **generalized additive mixed-effect models (GAMMs)**, a non-parametric, data-driven method that estimates non-linear relationships between predictors and outcome variables. This approach allowed us to estimate and visualize average changes in cognitive function throughout the disease trajectory, and to test how these trajectories differed between patients who reported loneliness and social isolation compared to a control group without such reports in their electronic health records (EHRs).

**GAMMs** are designed to model non-linear relationships between covariates and the dependent variable without assuming a specific parametric form, such as linear or quadratic. Instead of a fixed functional form, a **GAM** uses an unspecified smooth function, represented as:


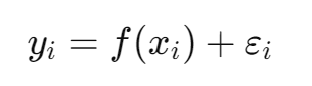


In this model, the function **f** is estimated through an iterative process, without predefining its shape. This makes GAMs **non-parametric** because they do not impose a specific form on the relationship between the predictor and outcome.

To estimate the non-linear effect, GAMs define a **basis function**—a space of functions that could represent **f**. For example, if we expect a relationship that follows a fourth-order polynomial, the model can explore a space that includes all polynomials up to that degree. The final relationship between the predictor and the dependent variable could then take the shape of, say, a sigmoidal curve.

Unlike standard linear models, where the functional form (e.g., polynomials or splines) must be predefined, **GAMs optimize the smooth function** iteratively, identifying the best-fitting relationship between the predictor and outcome.

#gam1<-bam(scal_score~CompNew+s(diag_dur_y, by=CompNew, bs='cs')+diag_cause+scale_age_y+sex+s(BRC_ID, bs='re'), data=moca2)

The formula used for the model is: scal_score ~ CompNew + s(diag_dur_y, by = CompNew, bs = "cs") + diag_cause + scal_age_y + sex + s(BRC_ID, bs = "re")

- scal_score: Longitudinal MoCa scores
- CompNew: A categorical variable indicating three report categories (Control, Isolation, Loneliness).
- s(diag_dur_y, by = CompNew, bs = "cs"): This term models the non-linear relationship between the duration of diagnosis (in years) and cognitive function, with different smooth functions for each category in CompNew.
- diag_cause: A categorical variable that represents the diagnosis cause (e.g., Alzheimer's, vascular dementia).
- scal_age_y: A continuous variable representing the age of the patient.
- sex: A binary variable for sex (Male/Female).
- s(BRC_ID, bs = "re"): This term models the random effect of BRC_ID, accounting for subject-specific variation, as patients are nested within a specific identifier.

gam1<-readRDS('Model1ThreeCateg_cleaned2.rds')

# Family: gaussian
# Link function: identity
#
# Formula:
# scal_score ~ CompNew + s(diag_dur_y, by = CompNew, bs = "cs") +
# diag_cause + scal_age_y + sex + s(BRC_ID, bs = "re")
#
# Parametric coefficients:
# Estimate Std. Error t value Pr(>|t|)
# (Intercept) 26.85474 0.96004 27.973 < 2e-16 ***
# CompNewIsolation -0.80431 0.26931 -2.987 0.00284 **
# CompNewLoneliness -0.89414 0.31635 -2.826 0.00472 **
# diag_causeDLB -0.18264 0.61258 -0.298 0.76560
# diag_causeMixedADVaD -0.55561 0.60617 -0.917 0.35940
# diag_causePPD -1.20823 0.85836 -1.408 0.15931
# diag_causeUnspecified 0.20467 0.18501 1.106 0.26868
# diag_causeVaD -1.60366 0.31031 -5.168 2.46e-07 ***
# scal_age_y -0.11544 0.01156 -9.984 < 2e-16 ***
# sexMale 1.06978 0.17272 6.194 6.34e-10 ***
# ---
# Signif. codes: 0 ‘***’ 0.001 ‘**’ 0.01 ‘*’ 0.05 ‘.’ 0.1 ‘ ’ 1
#
# Approximate significance of smooth terms:
# edf Ref.df F p-value
# s(diag_dur_y):CompNewNoComplaints 5.498 9 238.729 < 2e-16 ***
# s(diag_dur_y):CompNewIsolation 3.179 9 123.211 < 2e-16 ***
# s(diag_dur_y):CompNewLoneliness 2.242 9 76.312 8.99e-06 ***
# s(BRC_ID) 3660.018 4473 5.707 < 2e-16 ***
# ---
# Signif. codes: 0 ‘***’ 0.001 ‘**’ 0.01 ‘*’ 0.05 ‘.’ 0.1 ‘ ’ 1
#
# R-sq.(adj) = 0.756 Deviance explained = 85.9%
# fREML = 25666 Scale est. = 8.3974 n = 8700

**(Intercept):** The expected cognitive score for the reference category (Controls) at the baseline (average values of all other predictors).

**CompNewIsolation** and **CompNewLoneliness**: Both have significant negative effects on cognitive scores compared to the reference group (likely No Complaints). Isolation decreases the score by 0.80 points, while loneliness decreases it by 0.89 points.

**diag_causeVaD**: Vascular dementia (VaD) significantly decreases cognitive scores by 1.60 points.

**scal_age_y**: As age increases, cognitive scores decrease significantly by 0.12 points per year.

**sexMale**: Males tend to score 1.07 points higher than females, a significant effect.

**edf (effective degrees of freedom)**: The higher the edf, the more flexible (non-linear) the smooth function is. For example, s(diag_dur_y) has an edf of 5.498, suggesting a relatively complex non-linear relationship between diagnosis duration and cognitive scores for patients with no complaints.

**F:** This tests whether the smooth term adds significant explanatory power to the model.

**s(BRC_ID)**: The random effect for subject IDs is highly significant, indicating considerable variation across individuals that is captured by the model.

Key Takeaways:

- Social isolation and loneliness are both significantly associated with lower cognitive scores compared to those without such reports.
- Age has a negative impact on cognitive function, while sex (Male) shows a positive association.
- Patients with vascular dementia (VaD) show significantly lower cognitive function scores compared to other diagnostic causes.
- The non-linear relationship between diagnosis duration and cognitive function differs across groups (No Complaints, Isolation, Loneliness).
- There is significant variation at the individual level (captured by BRC_ID as a random effect).
- Overall, the model provides a comprehensive view of how cognitive function is influenced by multiple factors, including social isolation, loneliness, age, sex, and diagnostic causes. The non-linear effects of time since diagnosis are also important to understand the trajectory of cognitive decline.

Visualisations:

All groups:

plot_smooth(gam1, view='diag_dur_y', rm.ranef = F, se =1, plot_all='CompNew', col = c('darkviolet','darkgreen','darkblue'),xlab='Diagnosis duration (0 - time of diagnosis)', ylab='Cognitive functionality (MoCA)', ylim=c(13,20))


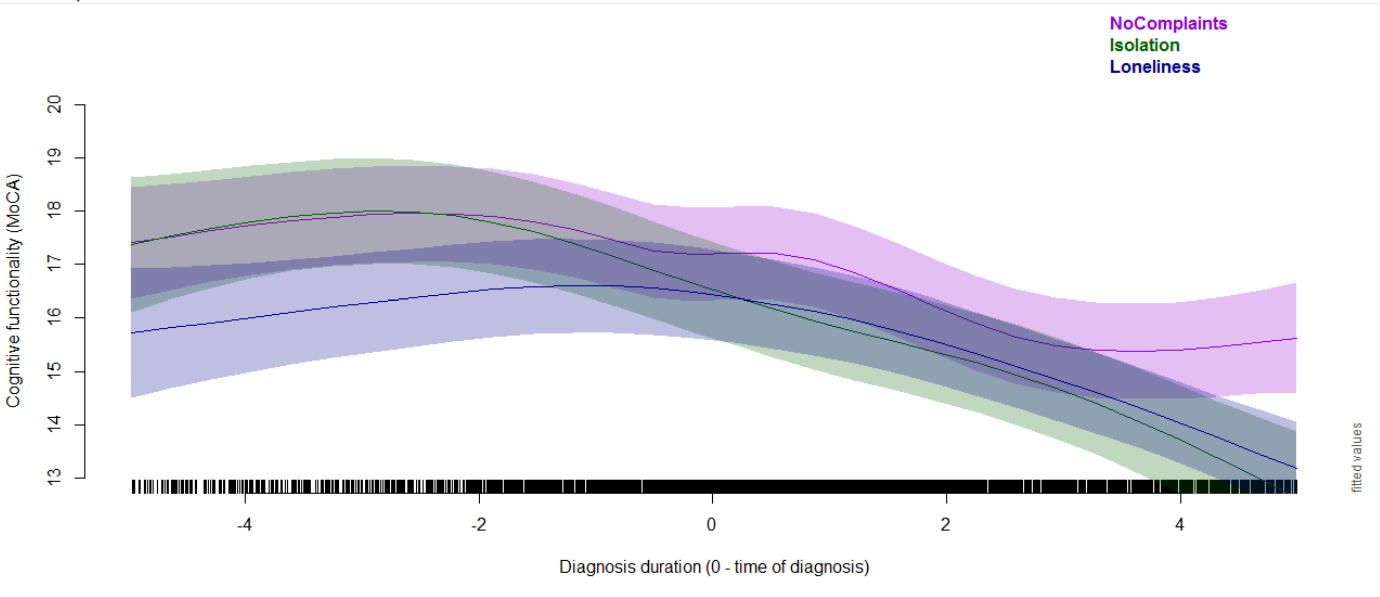


Effect of Loneliness:

Visualisation of differences between Control group (no reports) versus Loneliness group

par(cex=1.1, font.lab=2, font.axis=1.6, bty='n')
plot_smooth(gam1, view='diag_dur_y', rm.ranef = F, se =1, cond=list(CompNew='NoComplaints'), col = c('darkgreen'), xlab='Diagnosis duration (0 - time of diagnosis)', ylab='Cognitive functionality (MoCA)', ylim=c(12,20), rug=FALSE)

par(new=T)
plot_smooth(gam1, view='diag_dur_y', rm.ranef = F, se =1, cond=list(CompNew='Loneliness'), col = c('darkviolet'), xlab='', ylab='', ylim=c(12,20), rug=FALSE, lty=5)

legend(1.5,20, legend=c('Control','Loneliness reports'), col=c('darkgreen','darkviolet'),lty=c(1,5), cex=0.7)


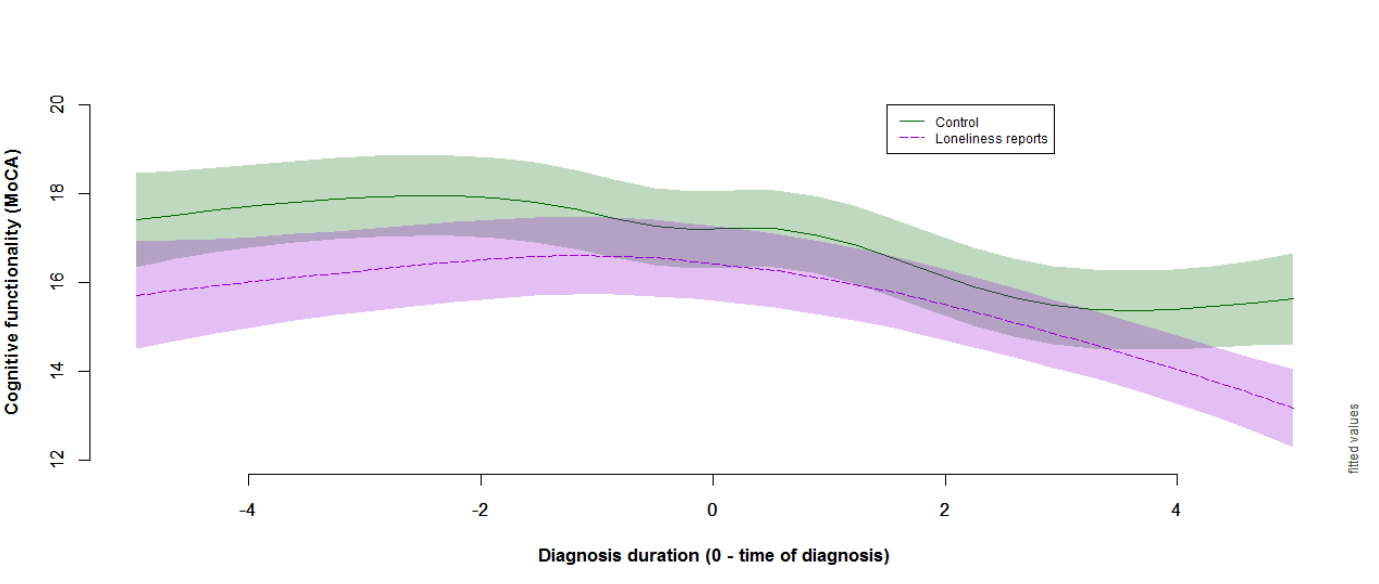


The plot_diff function from the **itsadug** package is used to visualize significant differences between the smooth curves estimated in a Generalized Additive Model (GAM). Specifically, it allows for the comparison of smooth splines for different levels of a factor or group, such as comparing how two groups (e.g., patients with and without reports of social isolation) differ over time or another continuous predictor.

Here's what the plot generated by plot_diff typically shows:

**Difference Between Smooth Curves**: The plot depicts the differences between two smooth curves over a continuous predictor variable (e.g., time, age, duration of illness). The y-axis represents the magnitude of the difference, and the x-axis represents the continuous predictor (e.g., time).

**Confidence Intervals**: Along with the estimated difference between the two curves, the plot includes confidence intervals (usually 95% confidence intervals). These are represented as shaded regions around the difference curve. The width of this region gives an idea of the uncertainty in the estimated difference at each point along the x-axis.

**Significance Threshold**: A reference line at zero is often shown, which indicates no difference between the two curves at that point. If the confidence interval crosses this zero line, it suggests that the difference between the two smooths is not statistically significant at that point. Conversely, if the confidence interval stays entirely above or below zero, it indicates a significant difference between the two smooth curves at that point on the x-axis.

**Direction of the Difference**:

If the difference curve is **above zero**, it indicates that the first group (e.g., patients without isolation) has higher values of the outcome variable compared to the second group (e.g., patients with isolation) for that specific value of the predictor.

If the difference curve is **below zero**, the second group has higher values for the outcome at that point.

par(cex=1.1, font.lab=2, font.axis=1.5, bty='n')
plot_diff(gam1, view='diag_dur_y', rm.ranef = F, se=1, comp=list(CompNew=c('NoComplaints','Loneliness')), ylab='Estimated difference in MoCA values', xlab='Diagnosis duration (0 - time of diagnosis)', main='Control minus Loneliness')


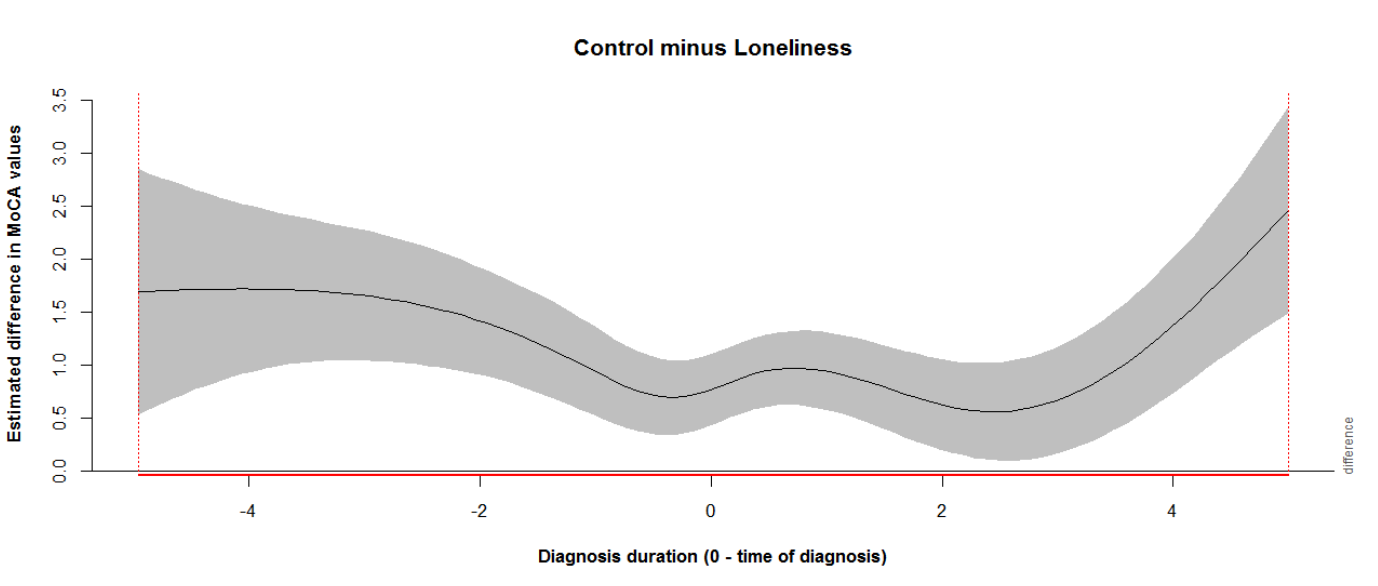


##
## diag_dur_y window(s) of significant difference(s):
## -4.958904 - 4.994521

**Fit of the model (residuals):**

When using the **draw()** function from the **gratia** package, it provides a graphical representation of the fitted GAM, including visualizations of the smooth terms, random effects, and residuals.

One of the most important outputs is the residual plot, which helps assess model fit and identify potential problems such as non-linearity, heteroscedasticity (unequal variance), or outliers.

X-axis: The fitted values (predicted values from the model).

Y-axis: The residuals (differences between observed and predicted values).

Interpretation:

Random scatter around zero indicates that the model is fitting well, with no obvious patterns left in the residuals.

Patterns or structure in the residuals (such as a funnel shape or a clear trend) indicate potential problems with the model fit, such as missing non-linear terms or heteroscedasticity.

Outliers: Extreme points that deviate far from the rest of the residuals suggest that there might be outliers in the data that the model is not handling well.

draw(gam1, residuals = TRUE)


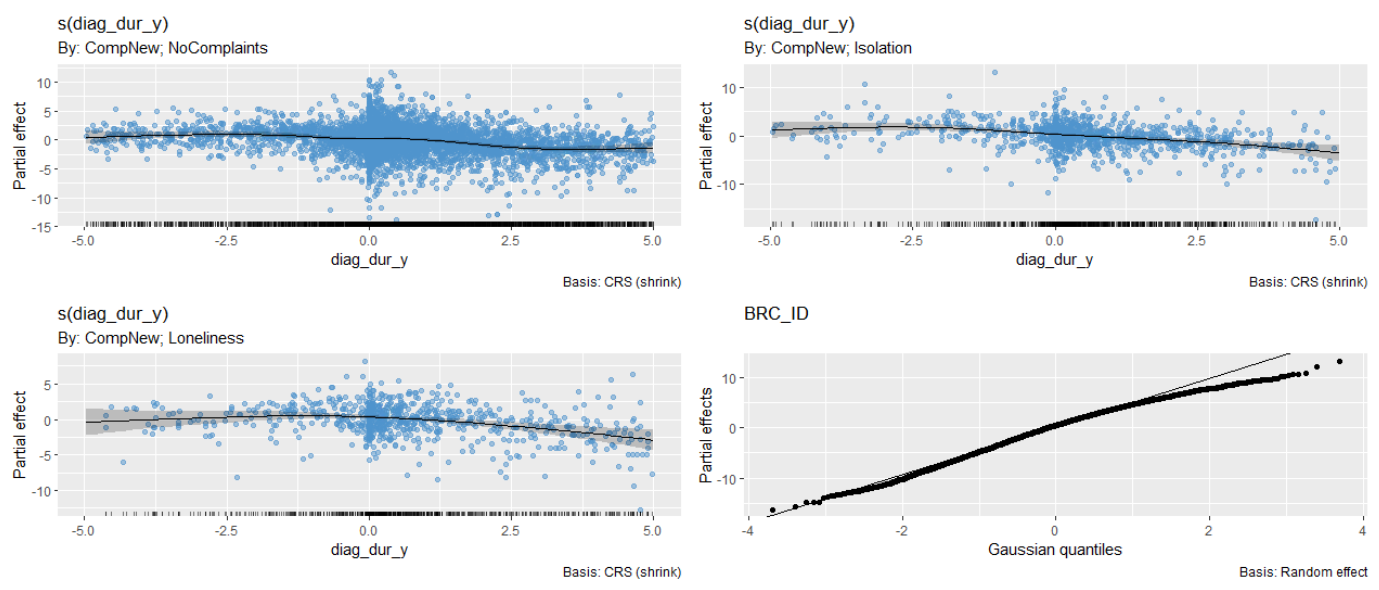


Linear mixed-effect models

We used linear mixed-effects regression (LMER) to model the data, implemented in the R statistical environment (Wood, 2017; R Core Team). This approach is particularly well-suited for controlling additional sources of variability in the dependent variable that are not directly influenced by the predictors.

When individual participants are treated as random effects, LMER provides estimates that balance between the overall group mean and each participant's individual data. This "shrinkage" effect pulls extreme values and participants with incomplete data toward the overall mean, reducing the influence of outliers and missing data (van Rij, Vaci, Wurm & Feldman, 2018).

Linear mixed-effects models are particularly valuable for analyzing repeated measures data, as they allow for the inclusion of multiple sources of variability in the dependent variable. They also handle data that may have non-Gaussian distributions or missing observations, making the analysis more robust in real-world settings.

**Unrestricted models:**

moca2$CompNew=as.factor(moca2$CompNew)
moca2$CompNew=relevel(moca2$CompNew, ref='NoComplaints')

lmerUn1<-lmer(scal_score ~ (1|BRC_ID), data=moca2)

lmerUn2<-lmer(scal_score ~ diag_dur_y + (1|BRC_ID), data=moca2)

lmerUn3<-lmer(scal_score ~ diag_dur_y + CompNew + (1|BRC_ID), data= moca2)

lmerUn4<-lmer(scal_score ~ diag_dur_y * CompNew + (1|BRC_ID), data=moca2)

anova(lmerUn1, lmerUn2, lmerUn3, lmerUn4)

## refitting model(s) with ML (instead of REML)

## Data: moca2
## Models:
## lmerUn1: scal_score ~ (1 | BRC_ID)
## lmerUn2: scal_score ~ diag_dur_y + (1 | BRC_ID)
## lmerUn3: scal_score ~ diag_dur_y + CompNew + (1 | BRC_ID)
## lmerUn4: scal_score ~ diag_dur_y * CompNew + (1 | BRC_ID)
## npar AIC BIC logLik deviance Chisq Df Pr(>Chisq)
## lmerUn1 3 55276 55297 -27635 55270
## lmerUn2 4 55081 55110 -27537 55073 196.4640 1 < 2.2e-16 ***
## lmerUn3 6 55063 55106 -27526 55051 21.5525 2 2.089e-05 ***
## lmerUn4 8 55064 55121 -27524 55048 3.8979 2 0.1424
## ---
## Signif. codes: 0 '***' 0.001 '**' 0.01 '*' 0.05 '.' 0.1 ' ' 1

summary(lmerUn3)

#### **
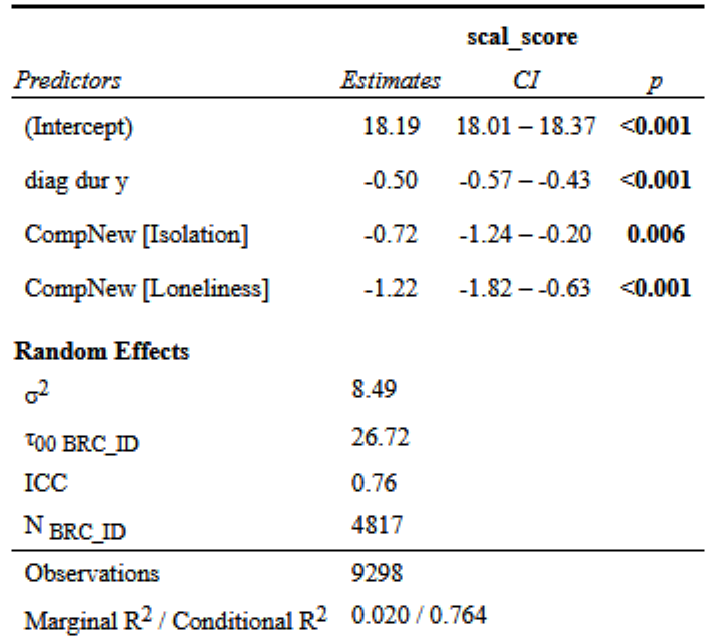
**

This linear mixed-effects model examines the relationship between cognitive score (scal_score), diagnosis duration (diag_dur_y), and the group variable CompNew, which represents patients with either reports of isolation, loneliness or controls, while accounting for random variability at the patient level (grouped by BRC_ID).

**1. Model Fit:**

REML Criterion: The model fit using Restricted Maximum Likelihood (REML) has a criterion value of 55060.9. This is a goodness-of-fit statistic that can be used for comparing models.

Residuals: The residuals are generally well-behaved, with the scaled residuals ranging from -4.92 to 4.20. Most residuals are small, with 1Q, Median, and 3Q values close to zero, indicating that the model fits most of the data reasonably well.

**2. Random Effects:**

The random effect of BRC_ID (i.e., individual patient IDs) has a variance of 26.72, with a standard deviation of 5.169. This means there is notable variability in cognitive scores between different patients.

The residual variance is 8.49 (with a standard deviation of 2.914), representing the remaining unexplained variability in the cognitive scores.

**3. Fixed Effects:**

The fixed effects capture the overall impact of diagnosis duration (diag_dur_y) and group membership (CompNew) on cognitive scores.

**Intercept (18.19, p < 2e-16):** The intercept is the baseline cognitive score for a patient with no reports of isolation or loneliness and a diagnosis duration of 0 years. This baseline score is 18.19.

**diag_dur_y (-0.50, p < 2e-16):** The coefficient for diagnosis duration indicates that for each additional year since diagnosis, cognitive scores decrease by approximately 0.50 points. This effect is highly significant, showing a clear relationship between longer diagnosis duration and lower cognitive function.

**CompNewIsolation (-0.72, p = 0.00632):** Patients with reports of isolation have cognitive scores that are, on average, 0.72 points lower than those with no reports. This effect is statistically significant, suggesting that isolation is associated with a modest but meaningful reduction in cognitive function.

**CompNewLoneliness (-1.22, p = 5.88e-05):** Patients with reports of loneliness have cognitive scores that are, on average, 1.22 points lower than those with no reports. This is a larger effect than isolation, and it is also highly significant, indicating that loneliness has a stronger negative impact on cognitive function compared to isolation.

4. **Overall Significance**:

- The model shows that both the duration of diagnosis and the experience of social isolation or loneliness significantly predict lower cognitive scores.
- Loneliness has a more pronounced negative effect than isolation, and both are detrimental compared to patients without such reports.

**Restricted models:**

In the next step, we added more controls to our models. Initially we modelled type of diagnosis, sex, and age, but then in separate analysis we added depressive symptomatology, accommodation and marital status.

lmerRe1<-lmer(scal_score ~ diag_dur_y + CompNew + diag_cause + (1|BRC_ID), data=moca2)

lmerRe2<-lmer(scal_score ~ diag_dur_y + CompNew + diag_cause + sex + (1|BRC_ID), data=moca2)

lmerRe3<-lmer(scal_score ~ diag_dur_y + CompNew + diag_cause + sex + scal_age_y + (1|BRC_ID), data=moca2)

lmerRe4<-lmer(scal_score ~ diag_dur_y * CompNew + diag_cause + sex + scal_age_y + (1|BRC_ID), data=moca2)

anova(lmerRe1, lmerRe2, lmerRe3, lmerRe4)

## refitting model(s) with ML (instead of REML)

## Data: moca2
## Models:
## lmerRe1: scal_score ~ diag_dur_y + CompNew + diag_cause + (1 | BRC_ID)
## lmerRe2: scal_score ~ diag_dur_y + CompNew + diag_cause + sex + (1 | BRC_ID)
## lmerRe3: scal_score ~ diag_dur_y + CompNew + diag_cause + sex + scal_age_y + (1 | BRC_ID)
## lmerRe4: scal_score ~ diag_dur_y * CompNew + diag_cause + sex + scal_age_y + (1 | BRC_ID)
## npar AIC BIC logLik deviance Chisq Df Pr(>Chisq)
## lmerRe1 13 51513 51605 -25744 51487
## lmerRe2 14 51464 51563 -25718 51436 51.2775 1 8.019e-13 ***
## lmerRe3 15 51371 51477 -25671 51341 94.6578 1 < 2.2e-16 ***
## lmerRe4 17 51371 51491 -25668 51337 4.5702 2 0.1018
## ---
## Signif. codes: 0 '***' 0.001 '**' 0.01 '*' 0.05 '.' 0.1 ' ' 1

summary(lmerRe3)

####
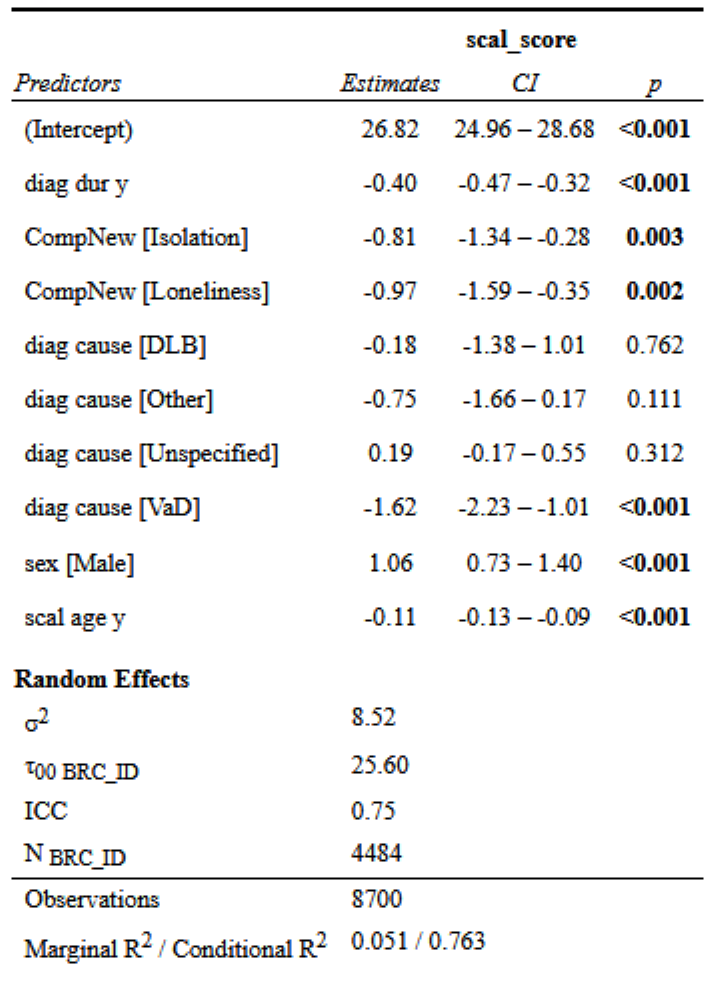


summary(lmerRe4)

###
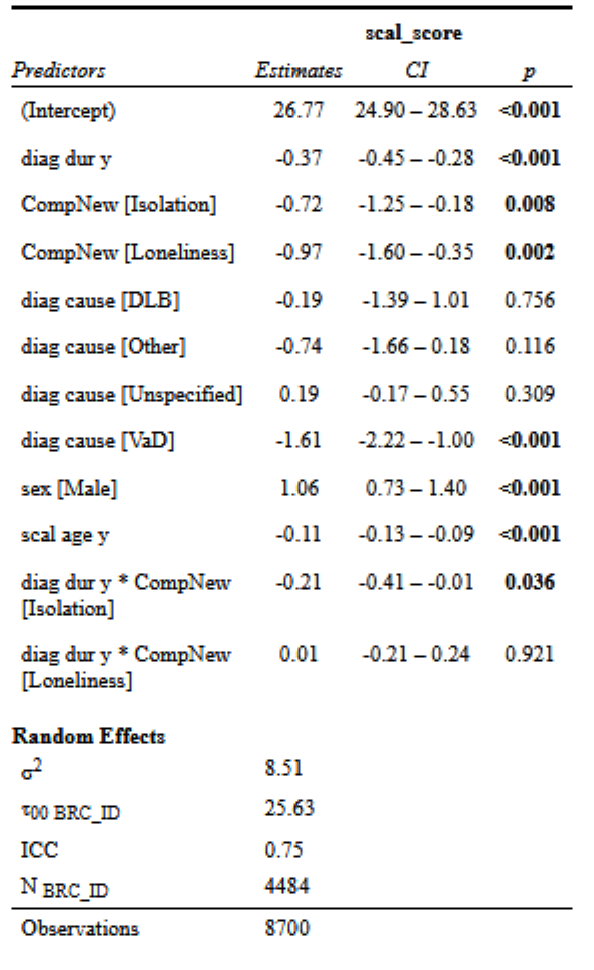


Additional controls

Depression:

lmerDep<-lmer(scal_score ~ diag_dur_y * CompNew + diag_cause + sex + scal_age_y + diagnosis_code+(1|BRC_ID), data=moca2)

summary(lmerDep)

####
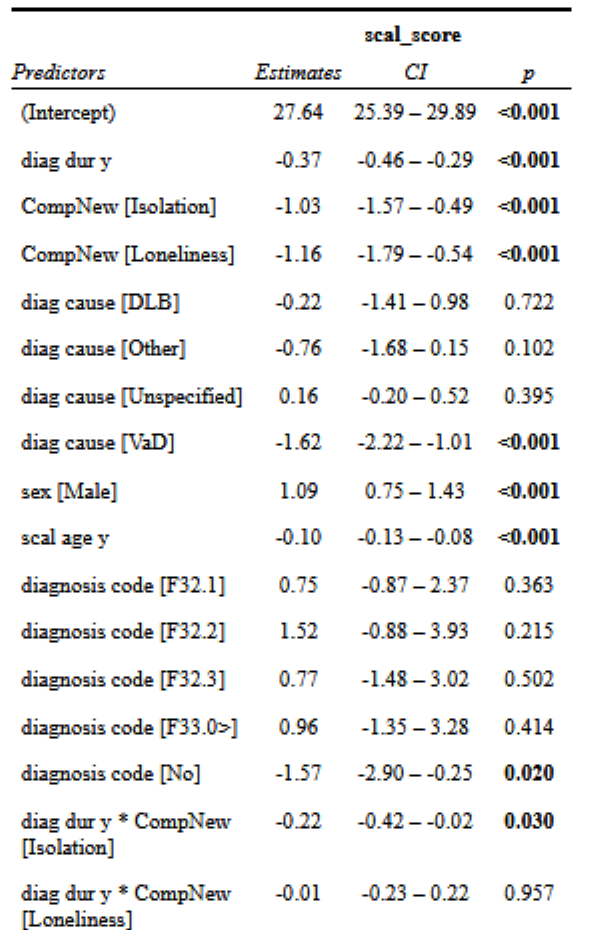


Marital status:

moca2$marital_status=ifelse(is.na(moca2$marital_status),'Missing',as.character(moca2$marital_status))

lmerMarital<-lmer(scal_score ~ diag_dur_y * CompNew + diag_cause + sex + scal_age_y + diagnosis_code+marital_status+(1|BRC_ID), data=moca2)

summary(lmerMarital)

####
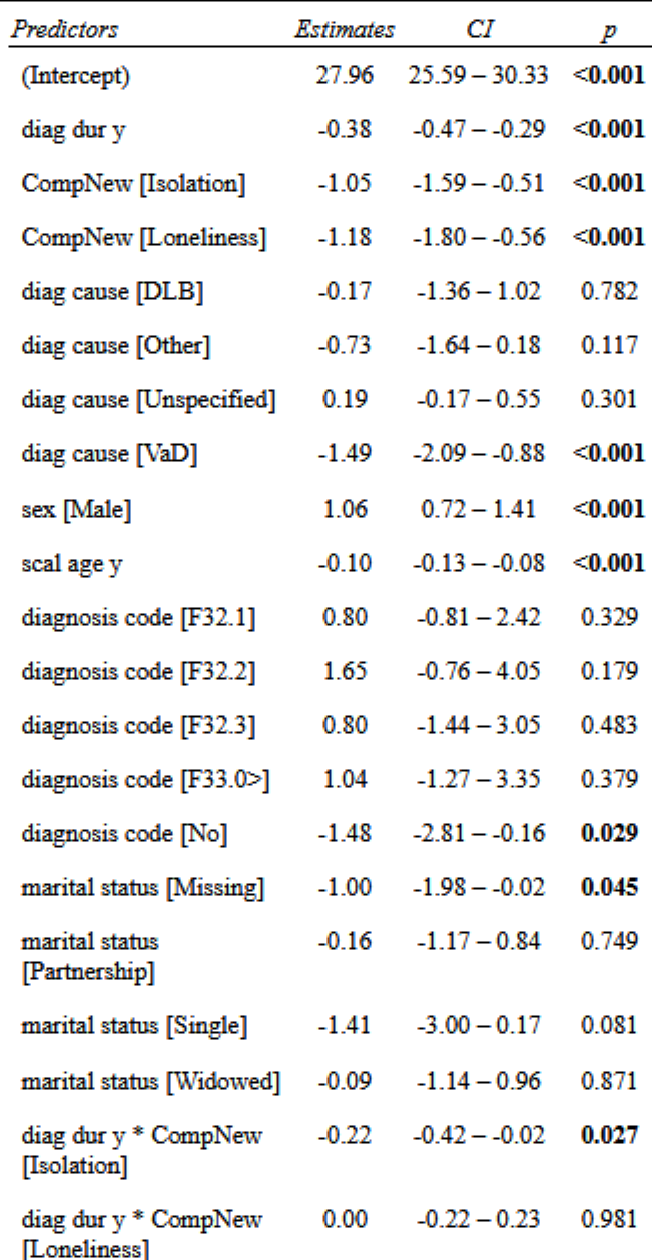


Accommodation status:

moca2$acco_status=ifelse(is.na(moca2$acco_status),'Missing',as.character(moca2$acco_status))

lmerAccommodation<-lmer(scal_score ~ diag_dur_y * CompNew + diag_cause + sex + scal_age_y + diagnosis_code+acco_status+(1|BRC_ID), data=moca2)

summary(lmerAccommodation)

####
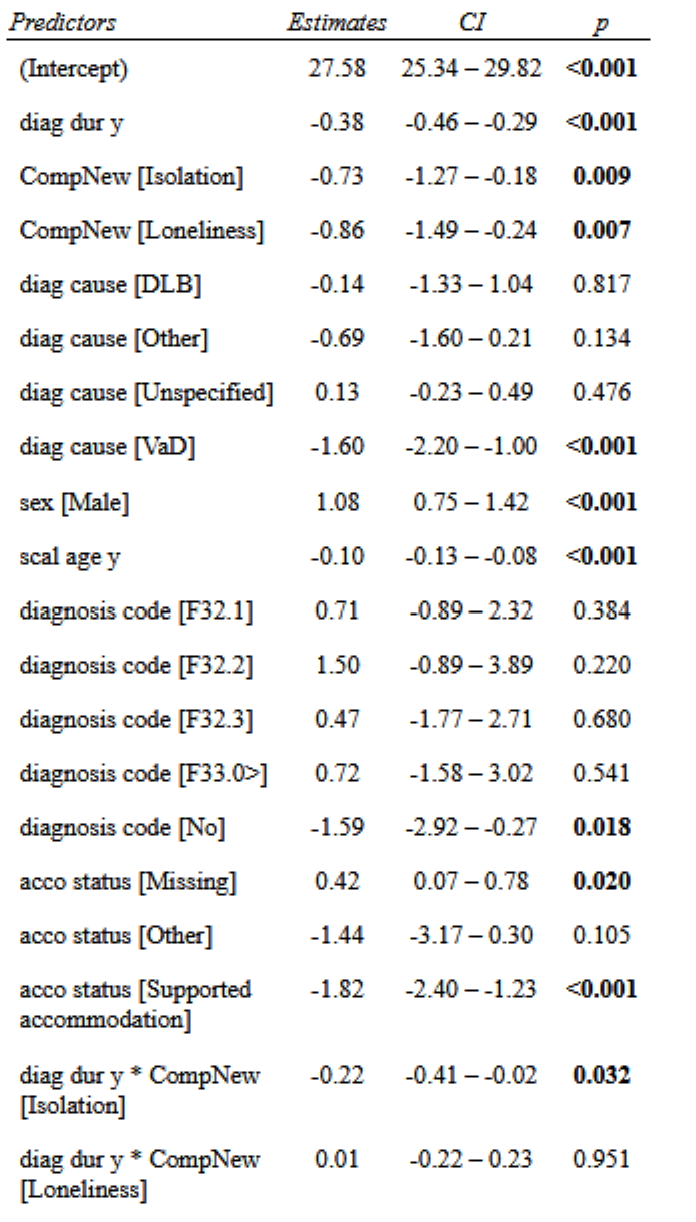


Joint controls:

lmerJoint<-lmer(scal_score ~ diag_dur_y * CompNew + diag_cause + sex + scal_age_y + diagnosis_code+marital_status+acco_status+(1|BRC_ID), data=moca2)

summary(lmerJoint)

##
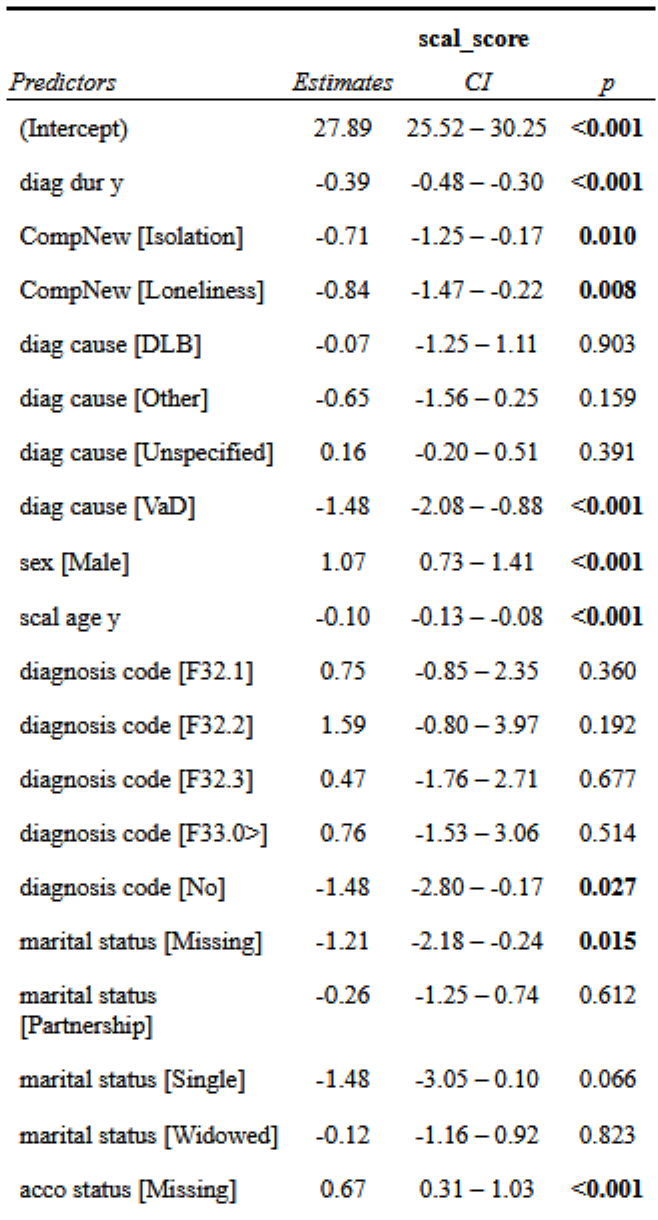


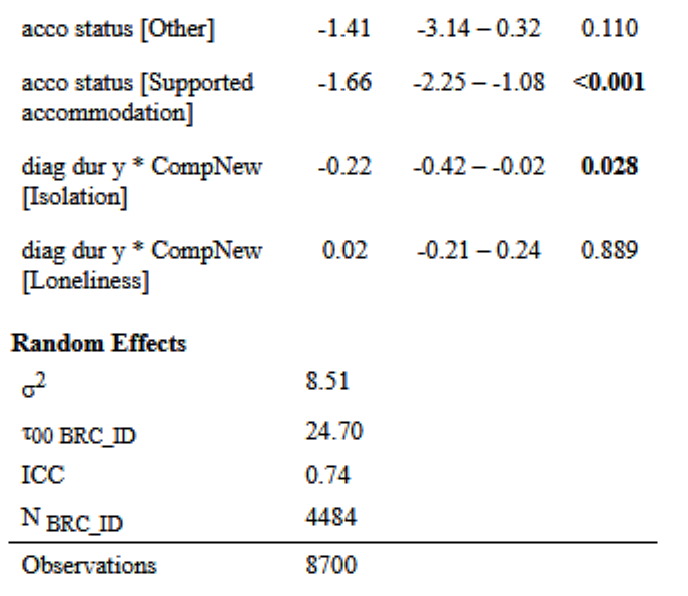


Change after the first complaint

In addition to modeling the association between social isolation, loneliness, and cognitive ability throughout the disease progression, we further investigated the rate of cognitive change after patients first reported social isolation or loneliness. Specifically, we analyzed the trajectory of cognitive decline relative to the time of the first complaint.

Timeline of Reports Relative to Diagnosis:

For both patient groups—those reporting social isolation and those reporting loneliness—we examined the timing of their complaints in relation to the date of their dementia diagnosis. Our analysis showed that the majority of patients reported issues with isolation or loneliness either before or at the time of their diagnosis. This finding indicates that social isolation and loneliness may often emerge or be recognized as concerns during the early stages of cognitive decline, which can coincide with the diagnostic process.

GAM Analysis of Cognitive Changes:

Using a **Generalized Additive Model (GAM)**, we visualized the average trajectories of cognitive change as measured by MoCA scores (Montreal Cognitive Assessment) in relation to the timing of their first complaints about social isolation or loneliness. We focused on changes in MoCA scores both before and after the reported issue, defining the time of the complaint as "time 0." This allowed us to capture and compare cognitive performance trends leading up to and following the onset of reported isolation or loneliness.

By plotting these trends, we were able to observe the overall decline in cognitive ability and how the rate of decline may have been influenced by the experience of isolation or loneliness. This provided insight into whether cognitive deterioration accelerates after patients begin to experience social challenges.

**Loneliness:**

moca3 = read.csv('LonelinessChangeAfterComp.csv')

moca3$diag_diff=ymd(moca3$diag_date)-ymd(moca3$DateComplaint)
moca3$diag_diff=as.numeric(moca3$diag_diff)

moca3 %>% ggplot(aes(x=diag_diff))+geom_histogram(color='#e9ecef', alpha = 2, position='identity')+ylab('Frequency')+xlab('Date of the first report relative to the date of diagnosis')


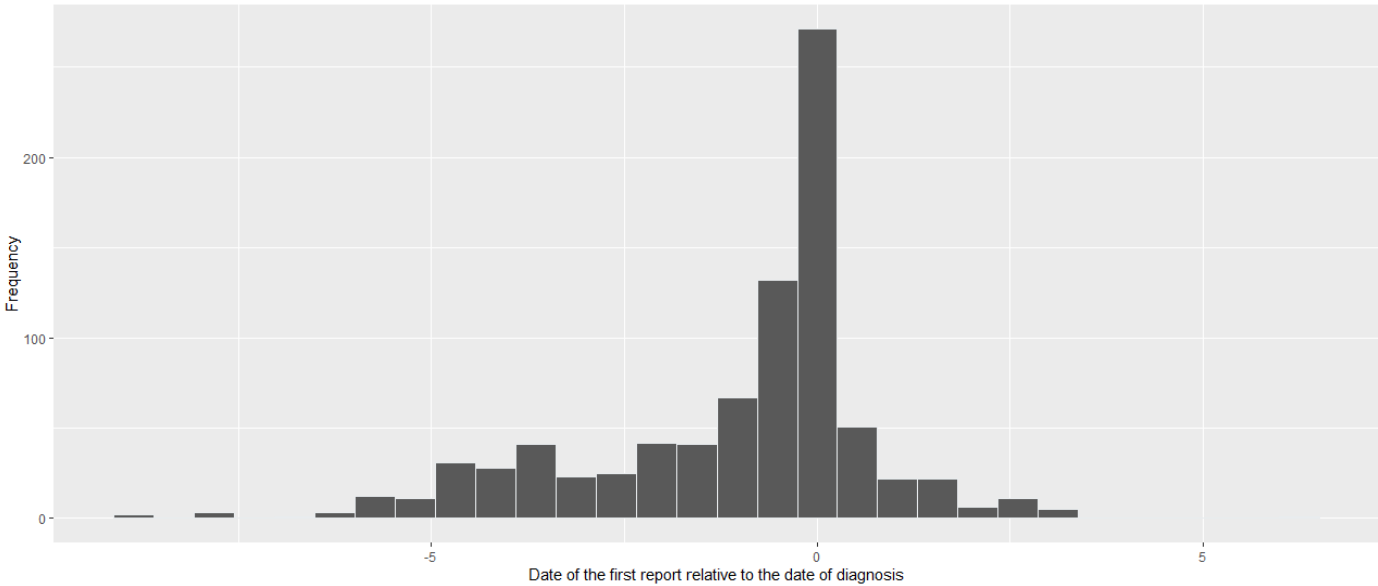


moca3$BRC_ID=as.factor(moca3$BRC_ID)

moca3$diff_comp=moca3$diff_comp/365

gam2<-bam(scal_score~ s(diff_comp)+s(diag_diff)+sex+scal_age_y+diag_cause+s(diff_comp,BRC_ID,bs='re', m=1), data=moca3)

par(cex=1.1, font.lab=2, font.axis=1.6, bty='n')
plot_smooth(gam2, view='diff_comp', se=1, ylab='Cognitive functionality (MoCA)', xlab='Timing of the first report (0 - report)', xlim=c(-3,3), ylim=c(14,21), main='Loneliness', rug=F)


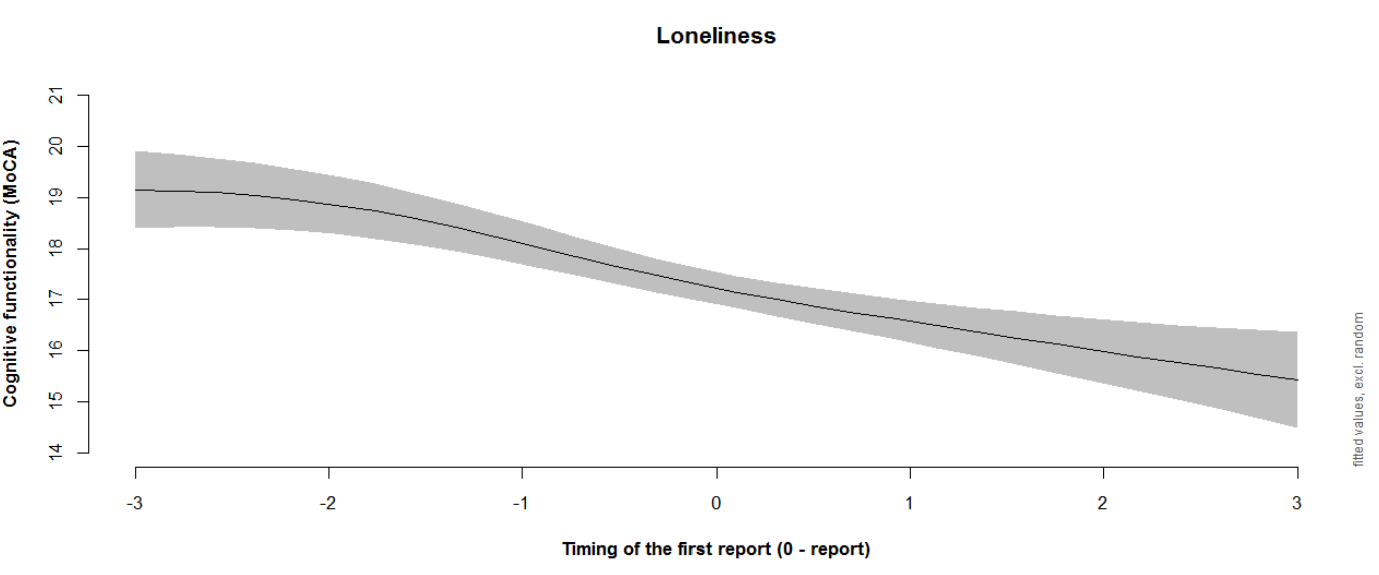


**Social Isolation:**

moca4 = read.csv('IsolationChangeAfterComp.csv')

moca4$diag_diff=ymd(moca4$diag_date)-ymd(moca4$DateComplaint)
moca4$diag_diff=as.numeric(moca4$diag_diff)

moca4 %>% ggplot(aes(x=diag_diff))+geom_histogram(color='#e9ecef', alpha = 2, position='identity')+ylab('Frequency')+xlab('Date of the measurement')

## `stat_bin()` using `bins = 30`. Pick better value with `binwidth`.


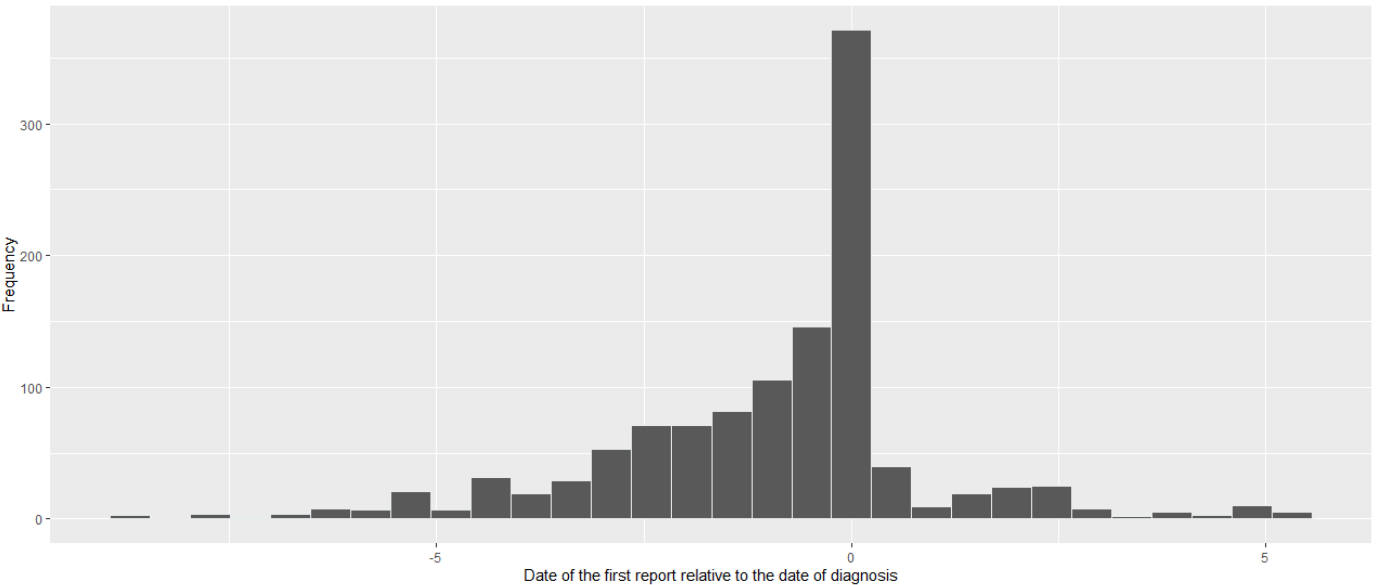


moca4$BRC_ID=as.factor(moca4$BRC_ID)

moca4$diff_comp=moca4$diff_comp/365

gam2<-bam(scal_score~ s(diff_comp)+s(diag_diff)+sex+scal_age_y+diag_cause+s(diff_comp,BRC_ID,bs='re', m=1), data=moca4)

par(cex=1.1, font.lab=2, font.axis=1.6, bty='n')
plot_smooth(gam2, view='diff_comp', se=1, ylab='Cognitive functionality (MoCA)', xlab='Timing of the first report (0 - report)', xlim=c(-3,3), ylim=c(14,21), main='Isolation')


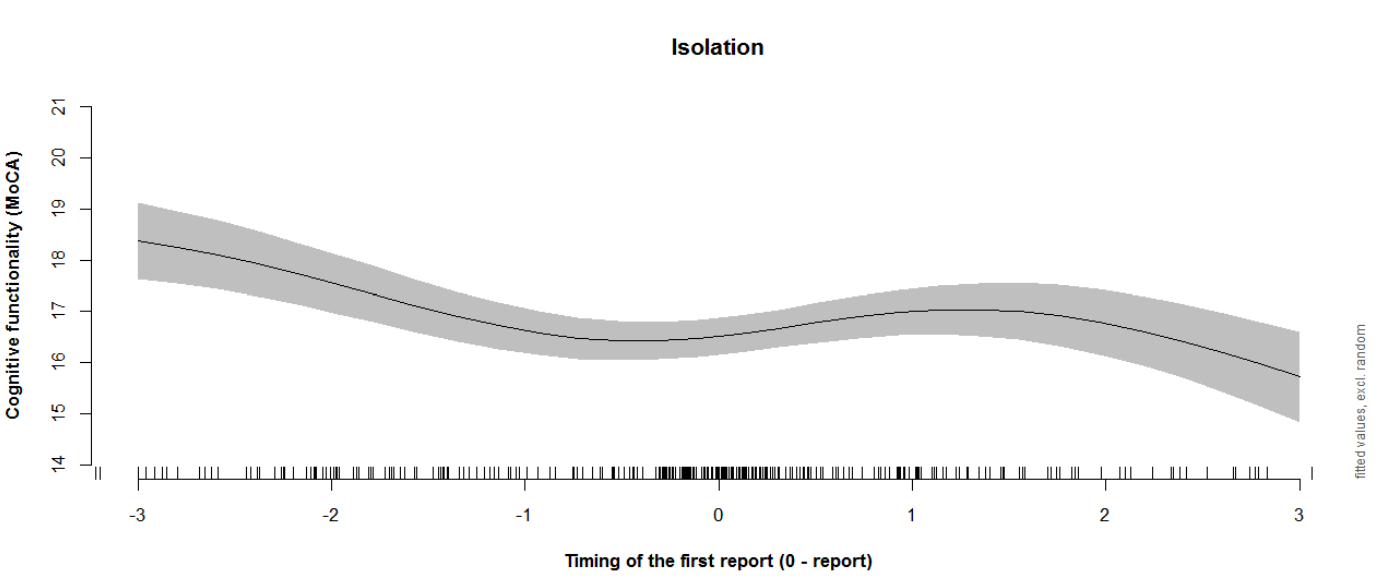


Age effects

age<-read.csv('AgeDiagnosis.csv')

age$CompNew=as.factor(age$CompNew)
age$CompNew=relevel(age$CompNew, ref='NoComplaints')

lm1<-lm(age_first_obs~CompNew, data=age)

summary(lm1)


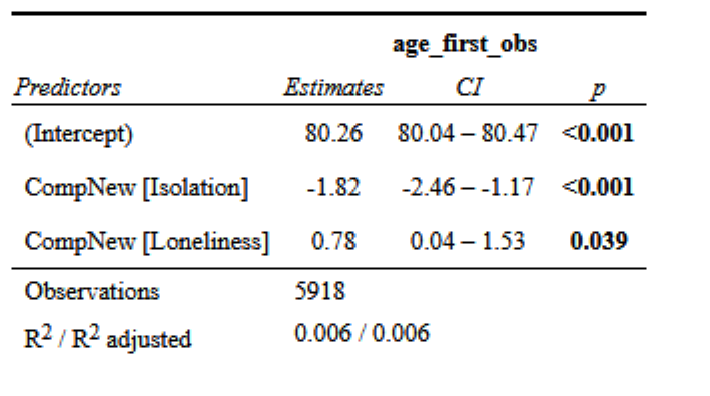


lm2<-lm(age_diag_y~CompNew, data=age)
summary(lm2)

**
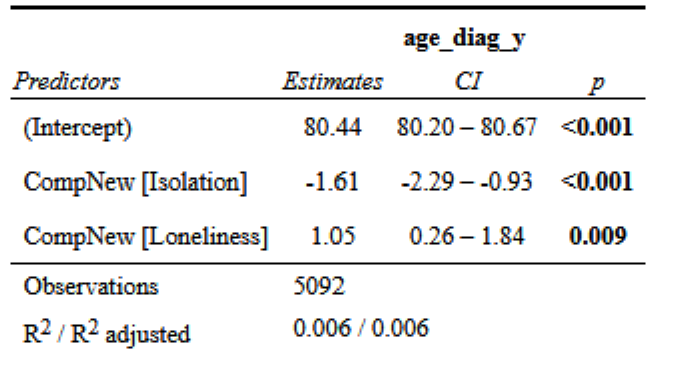
**
