# Supplementary material for: Loneliness, social isolation, and effects on cognitive decline in patients with dementia: A retrospective cohort study using natural language processing
Source: Alzheimers Dement (Amst). 2025 Jul 28;17(3):e70149. doi: 10.1002/dad2.70149 (PMC12301694; doi:10.1002/dad2.70149)

Supplemental materials for Integrating Natural Language Processing to Assess Effect of Loneliness and Social isolation on Cognitive Decline in Dementia: A Retrospective Cohort Analysis

James Myers^1,2,9,10^, Tom Stafford^1,2^, Ivan Koychev^3,9^, Robert Perneczky^2,4,5,6,7,8^, Oliver Bandmann^2,4^, Nemanja Vaci^1,2,9,10^*

1. School of Psychology, University of Sheffield, UK
2. Division of Neuroscience, University of Sheffield, Sheffield, UK
3. Department of Psychiatry, University of Oxford, UK
4. School of Medicine and Population Health, University of Sheffield, UK
5. Department of Psychiatry and Psychotherapy, LMU Hospital, LMU Munich, Munich, Germany
6. German Center for Neurodegenerative Diseases (DZNE), Munich, Germany
7. Munich Cluster for Systems Neurology (SyNergy), Munich, Germany
8. Ageing Epidemiology Research Unit (AGE), School of Public Health, Imperial College London, London, UK
9. Oxford Health NHS Foundation Trust
10. Rotherham, Doncaster and South Humber NHS Foundation Trust

| **Table of Contents** | Page |
| --- | --- |
| **N**LP model: social isolation and loneliness | 3 |
| NLP Accuracy | 6 |
| Sensitivity Analysis |  |
| Missing data handling | 10 |
| Timing of report | 13 |
| MMSE analysis | 16 |
| Patients with reports of social isolation and loneliness | 23 |

N**LP model: social isolation and loneliness**

The Natural Language Processing (NLP) model processed mentions of social isolation and loneliness in two key stages.

**Step 1: Document Scanning and Filtering**

In the first stage, the model scanned the full medical documents for any mentions of social isolation and loneliness using Spacy small English language model (en_core_web_sm). When these mentions were identified, the model extracted the relevant sentences, recording whether the mention was negated (i.e., cases where the patient was noted as not experiencing social isolation or loneliness). Sentences with affirmed reports of social isolation and loneliness were then filtered for further analysis.

**Step 2: Classification Using Sentence Transformers**

In the second stage, we employed sentence transformers from Huggingface’s spacy-setfit library (<https://github.com/davidberenstein1957/spacy-setfit>) to categorize the extracted reports. **Sentence Transformers** are a type of neural network model designed to produce dense vector representations (embeddings) of entire sentences or text fragments. These models are particularly useful for tasks where the semantic meaning of a sentence needs to be captured in a fixed-length vector, making them powerful for tasks like sentence similarity, clustering, and classification. Unlike traditional word embeddings (e.g., Word2Vec), which represent individual words, sentence transformers generate embeddings that capture the meaning of an entire sentence. These embeddings encode semantic relationships, allowing similar sentences to have similar vector representations, even if the wording is different. Sentence transformers are based on the transformer architecture (e.g., BERT, RoBERTa), which uses self-attention mechanisms to process sequences of words. This allows the model to consider the context of each word in the sentence, leading to better understanding of complex language structures. While pretrained sentence transformers are powerful, they can be fine-tuned on specific datasets to improve performance on specialized tasks like text classification, sentiment analysis, or question answering. Fine-tuning allows the model to adapt to the nuances of the target domain.

The transformer model was trained to classify the sentences into one of four categories:

1. **Social isolation reports**
2. **Loneliness reports**
3. **Non-informative isolation reports** (mentions that lack meaningful context)
4. **Non-informative sentences** (sentences irrelevant to social isolation or loneliness)

Python code for the NLP model is provided below. We provide examples of the sentences used to train the model at the end of the code block.

import spacy
from spacy.matcher import Matcher
from spacy.language import Language
from spacy.tokens import Doc
import pickle
from spacy.tokens import Span


nlp = spacy.load("en_core_web_sm")

lon_patterns = [
 [{"LOWER":"loneliness"}],
 [{"LOWER": "lonely"}],
 [{"LOWER": "isolated"}],
 [{"LOWER": "social"},{"LOWER": "isolation"}],
 [{"LOWER": "isolated"},{"LOWER": "from"},{"LOWER": "society"}],
 [{"LOWER": "socially"},{"LOWER": "withdrawn"}],
 [{"LOWER": "living"},{"LOWER": "alone"}],
 [{"LOWER": "no"},{"LOWER": "social"},{"LOWER": "support"}],
 [{"LOWER": "nobody"},{"LOWER": "to"},{"LOWER": "talk"}],
 [{"LOWER": "being"},{"LOWER": "so"},{"LOWER": "alone"}],
 [{"LOWER": "feel"},{},{"LOWER": "alone"}],
 [{"LOWER": "feel"},{"LOWER": "left"},{"LOWER": "out"}],
 [{"LOWER": "starved"},{"LOWER": "for"},{"LOWER": "company"}]
]

---
@Language.component('loneliness_component')
def loneliness_component(doc):
 matcher = Matcher(nlp.vocab)
 matcher.add("LONELINESS",lon_patterns)
 matches = matcher(doc)

 if not Doc.has_extension("loneliness_spans"):
 Doc.set_extension("loneliness_spans", default=[])
 doc._.loneliness_spans = [doc[start:end] for _, start, end in matches]
 return doc


 @Language.component('negation_component')

---
def negation_component(doc):
 negation_terms = {"not","no","never",'does not','doesn\'t'}

 if not Doc.has_extension("loneliness_spans"):
 raise ValueError('Run loneliness matcher first')

 if not Doc.has_extension("negation_detected"):
 Doc.set_extension("negation_detected", default =[])
 doc._.negation_detected = []

 for loneliness_span in doc._.loneliness_spans:
 negation_found = False
 negation_span = []
 start = max(loneliness_span.start - 3, 0)
 end = min(loneliness_span.end + 3, len(doc))
 surounding_tokens = doc[start:end]

 for token in surounding_tokens:
 if token.text.lower() in negation_terms:
 negation_span.append(token.text)
 break
 doc._.negation_detected.append(negation_span)
 return doc

---

@Language.component('sentence_filter')
def sentence_filter(doc):
 selected_sentences = []
 for sent in doc.sents:
 if any(loneliness_span.start >= sent.start and loneliness_span.end <= sent.end for loneliness_span in doc._.loneliness_spans):
 selected_sentences.append(sent)
 if not Doc.has_extension('selected_sentences'):
 Doc.set_extension('selected_sentences',default=[])
 doc._.selected_sentences = selected_sentences
 return doc

nlp.add_pipe("loneliness_component", last=True)
nlp.add_pipe("negation_component", last = True)
nlp.add_pipe("sentence_filter", last = True)

for index, row in df2.iterrows():
 doc = nlp(row['Clinical_Note_Text'])
 df2.at[index,"matched_terms"] = str(doc._.loneliness_spans)
 df2.at[index,"negation"] = str(doc._.negation_detected)
 df2.at[index,'sentence'] = str(doc._.selected_sentences)

data = {
 "Isolation" : [

"Due to XXXXXs impairments and symptoms, she has gradually isolated from others”,

"Resides alone in her own home.”,

"XXX experiences social isolation, currently living in 3^rd^ floor flat with no lift”,

"Increase in isolation due to no longer driving”,

"XXXXX feels his parent is more distressed and isolated and frightened”],

 "Loneliness": [

'Reports feeling lonely but is not trying to change this’,

'XXXXX his parent becomes lonely at times’,

"Lonely and without any friends”,

"Initial referral with anxiety and loneliness",
 ],

 "Random": [

"She has also complained of feeling dizzy when standing from a seated position

"XXX will be discharged from memory clinic back to your care.”]

 "NegativeIso": ['On the morning of the event was feeling isolated',
 "Alone in the tv lounge as wanting some peace ",
 "this is currently an isolated incidence",
 "has suffered an isolated fall",
 ]}

nlp.add_pipe("spacy_setfit", config={
 "pretrained_model_name_or_path" : "transformers/sentence-transformers--all-MiniLM-L6-v2.main.44eb4044493a3c34bc6d7faae1a71ec76665ebc6",
 "setfit_trainer_args": {
 "train_dataset": data,
 "num_iterations": 30,
 "num_epoch": 3,
 "learning_rate": 2e-5
 }
})

Example of processing:

text= 'He is lonely, almost every day, not in contact with family'
doc = nlp(text)
doc.cats
{'Isolation': 0.04865152182010921,
 'Loneliness': 0.8872721353900636,
 'Random': 0.02313443634662194,
 'NegativeIso': 0.04094190644320528}

NLP Accuracy

The model was trained iteratively on a subset of 11,000 randomly selected medical documents, refining its accuracy through multiple rounds of training. Once satisfactory classification performance was achieved, it was tested on an unseen set of 5,000 documents to validate its accuracy and generalization. The 5,000 documents used to test the final performance of the model were separately annotated by the first and last author, while in the case of categorisation disagreement they discussed final annotations and augmented them to reflect the joint agreement.

We evaluated the performance of our NLP classifier by comparing its predictions to manually annotated documents. The annotations categorized sentences into five distinct categories: **Isolation**, **Loneliness**, **Non-informative Isolation**, and **Non-informative sentence**. The aim of this evaluation was to determine how accurately the classifier could distinguish between these categories based on the annotations.

Confusion Matrix Construction

For each category, we constructed a **confusion matrix** that represents the classifier’s performance. The confusion matrix provides a breakdown of true positives (TP), true negatives (TN), false positives (FP), and false negatives (FN) for each category:

- **True Positives (TP)**: Instances correctly predicted as belonging to a specific category.
- **True Negatives (TN)**: Instances correctly predicted as not belonging to the category.
- **False Positives (FP)**: Instances incorrectly predicted as belonging to the category.
- **False Negatives (FN)**: Instances incorrectly predicted as not belonging to the category.

From this matrix, we calculated a range of performance metrics, both at the category level and overall.

Performance Metrics Calculated

**Sensitivity (Recall or True Positive Rate)**:


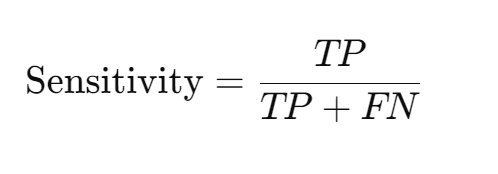


Sensitivity measures the proportion of actual positive instances that are correctly identified by the classifier. It indicates how well the model identifies a given category when it is truly present.

**Specificity (True Negative Rate)**:


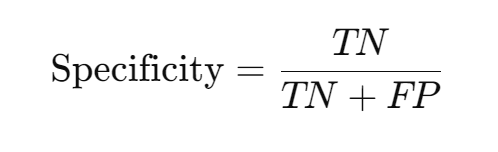
 ​

Specificity measures the proportion of actual negative instances that are correctly identified. It reflects how well the model avoids false positives for a category.

**Positive Predictive Value (PPV or Precision)**:


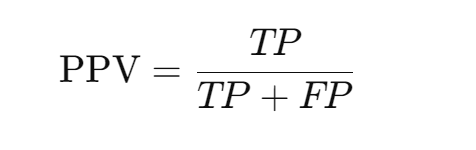
 ​

Positive Predictive Value indicates the proportion of instances classified as positive that are actually positive. This metric is especially important when dealing with imbalanced categories.

**Negative Predictive Value (NPV)**:


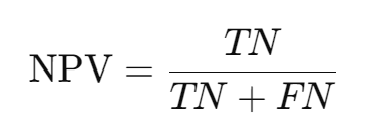


Negative Predictive Value measures the proportion of instances classified as negative that are truly negative, providing insight into the classifier’s reliability in predicting non-positives.

**Prevalence**:


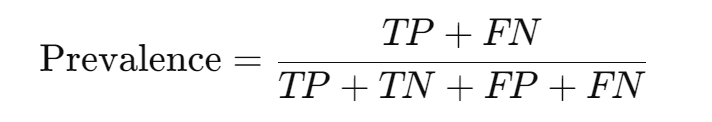


Prevalence represents the proportion of instances in the dataset that actually belong to the positive category.

**Detection Rate**:


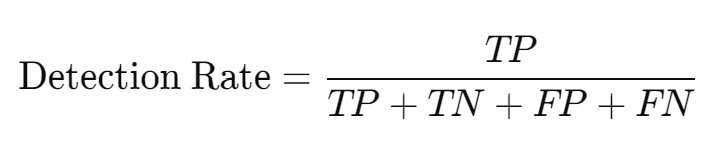


The Detection Rate provides the overall rate at which the classifier correctly detects the positive instances in the entire dataset.

**Detection Prevalence**:


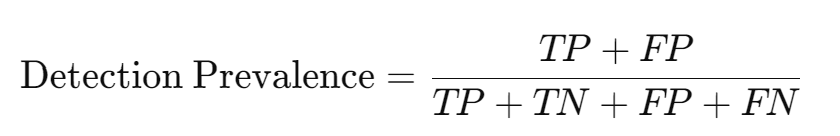
 ​

Detection Prevalence is the proportion of instances the classifier predicts as belonging to the positive class.

**Balanced Accuracy**:


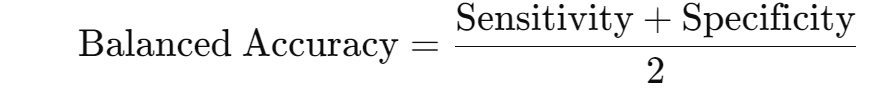
 ​

Balanced Accuracy provides an average measure of the classifier’s performance across both positive and negative classes, accounting for any imbalances in the dataset.

NLPacc=read.csv('ModelPerformance_v0_s2.csv')
NLPacc$Agreement=as.factor(NLPacc$Agreement)
levels(NLPacc$Agreement)=c('Isolation','Isolation','Loneliness','Negative','Negative','Random','Random')

NLPacc$IsoCat=ifelse(NLPacc$Isolation>=0.50,1,0)
NLPacc$LonCat=ifelse(NLPacc$Loneliness>=0.50,1,0)
NLPacc$RandomCat=ifelse(NLPacc$Random>=0.50,1,0)
NLPacc$NegativeCat=ifelse(NLPacc$NegativeIso>=0.50,1,0)

NLPacc$Prediction=ifelse(NLPacc$IsoCat==1,'Isolation', ifelse(NLPacc$LonCat==1,'Loneliness',ifelse(NLPacc$RandomCat==1,'Random',ifelse(NLPacc$NegativeCat==1,'Negative','Random'))))

NLPacc$Prediction=as.factor(NLPacc$Prediction)

levels(NLPacc$Agreement)=levels(NLPacc$Prediction)

confusionMatrix(NLPacc$Prediction, NLPacc$Agreement)

| **Reference → Prediction** | **Isolation** | | **Loneliness** | | **Negative** | **Random** |
| --- | --- | --- | --- | --- | --- | --- |
| Isolation | 19 | | 2 | | 1 | 1 |
| Loneliness | 0 | | 23 | | 2 | 1 |
| Negative | 0 | | 0 | | 0 | 3 |
| Random | 7 | | 1 | | 3 | 24 |
|  |  | |  | |  |  |
| **Overall Statistics:** | | | |  |  |  |
|  | |  | |  |  |  |
| **Metric** | | **Value** | |  |  |  |
| Accuracy | | 0.7586 | |  |  |  |
| 95% CI | | (0.655, 0.844) | |  |  |  |
| No Information Rate | | 0.3333 | |  |  |  |
| P-Value [Acc > NIR] | | 5.82E-16 | |  |  |  |
| Kappa | | 0.6528 | |  |  |  |
| McNemar's Test P-Value | | 0.1473 | |  |  |  |

| **Statistics by Class:** | |  |  |  |
| --- | --- | --- | --- | --- |
|  |  |  |  |  |
| **Metric** | **Isolation** | **Loneliness** | **Negative** | **Random** |
| Sensitivity | 0.7308 | 0.8846 | 0 | 0.8276 |
| Specificity | 0.9344 | 0.9508 | 0.96296 | 0.8103 |
| Pos Pred Value | 0.8261 | 0.8846 | 0 | 0.6857 |
| Neg Pred Value | 0.8906 | 0.9508 | 0.92857 | 0.9038 |
| Prevalence | 0.2989 | 0.2989 | 0.06897 | 0.3333 |
| Detection Rate | 0.2184 | 0.2644 | 0 | 0.2759 |
| Detection Prevalence | 0.2644 | 0.2989 | 0.03448 | 0.4023 |
| Balanced Accuracy | 0.8326 | 0.9177 | 0.48148 | 0.819 |

**Sensitivity Analysis**

In the main analysis presented in the manuscript, we used indicator variable as missing data handling approach. In particular, all missing observations were modelled as an additional level “Missing”, allowing us to keep all observations when estimating the model. However, this might bias the estimates. Therefore we included sensitivity analysis, where we first analyse the data without predictors with large number of missing observations and secondly model with case-wise deletion of patients with missing data.

**Model without accommodation and marital status predictors:**


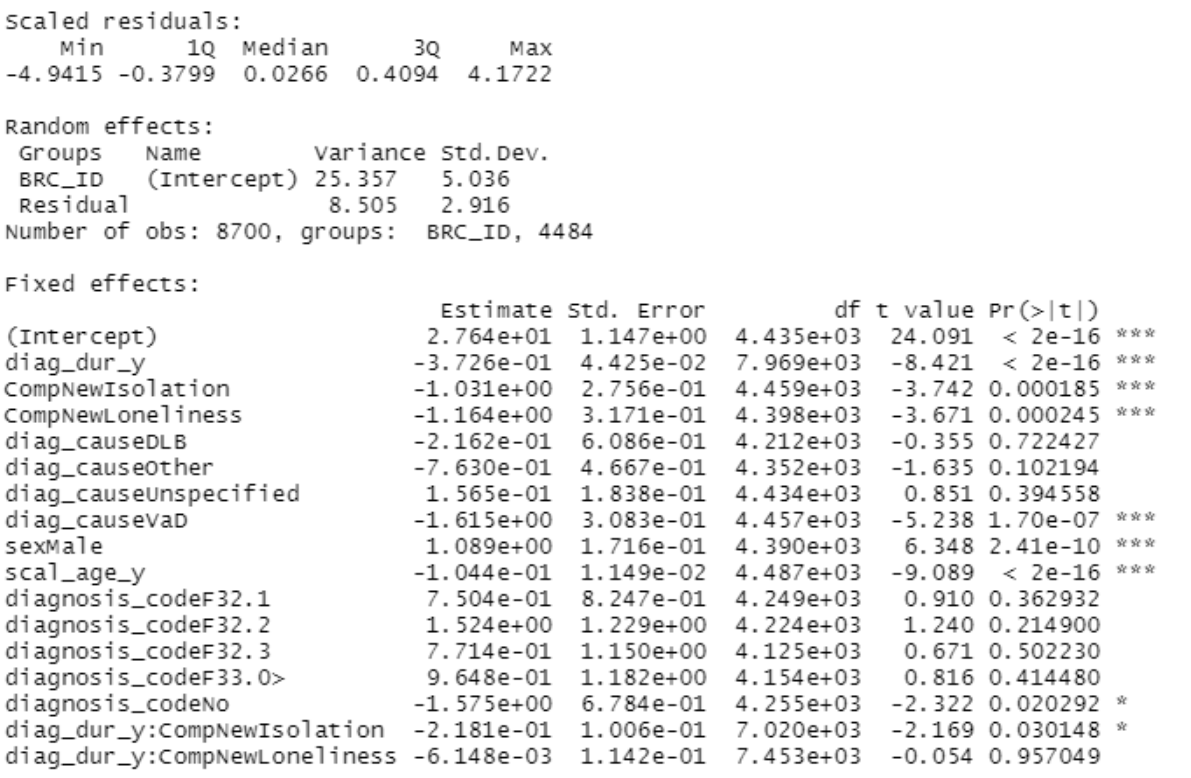


Results show identical patterns of change in MoCA scores as in the case of main analysis.

**Model without missing observations for accommodation predictor:**

**
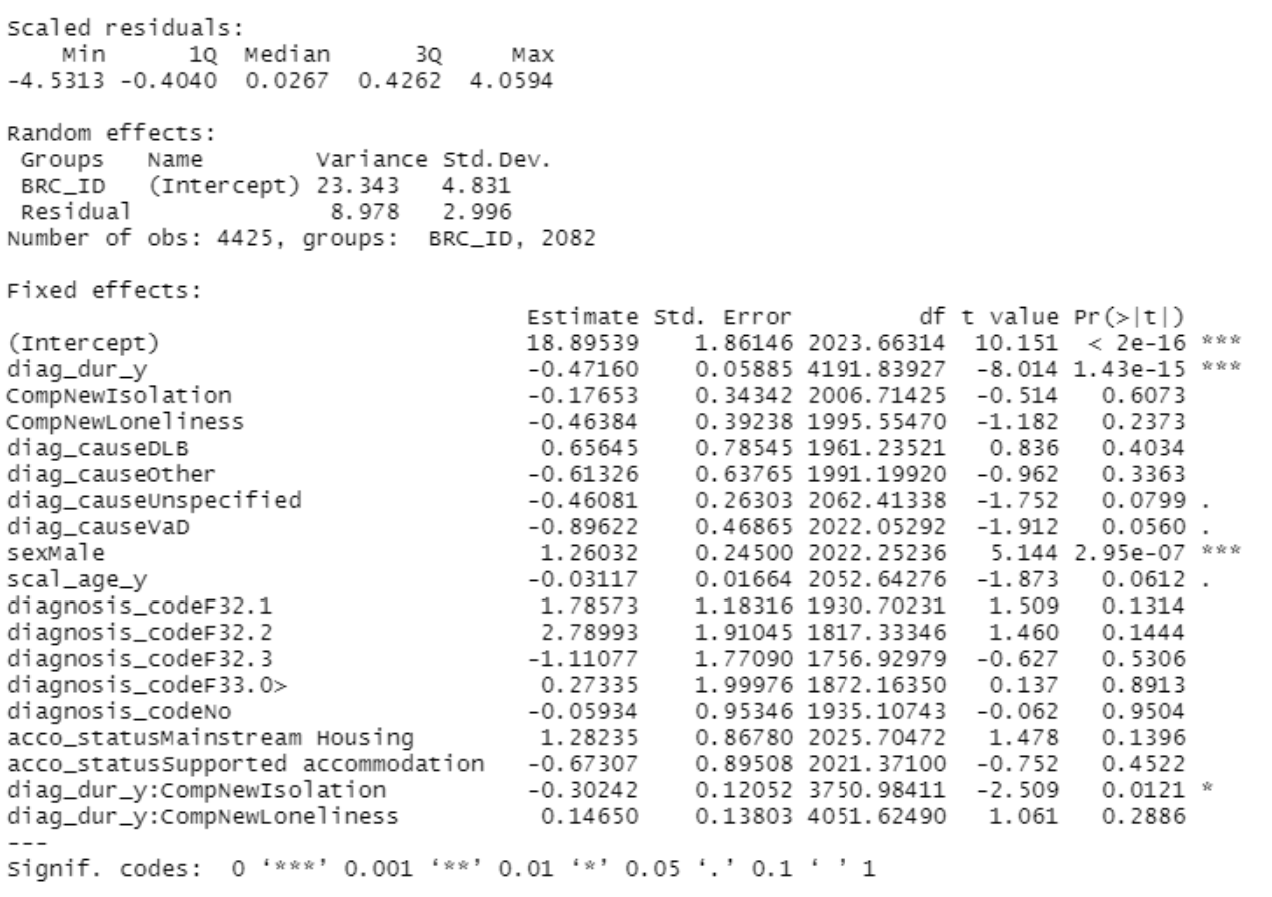
**

When excluding all patients with missing observation for accommodation status, we lose more than 50% of patients and their MoCA scores.

The trends are replicated, but only changes in the slope across the disease duration for Socially isolated group stay significant.

**Model without missing observations for marital status predictor:**
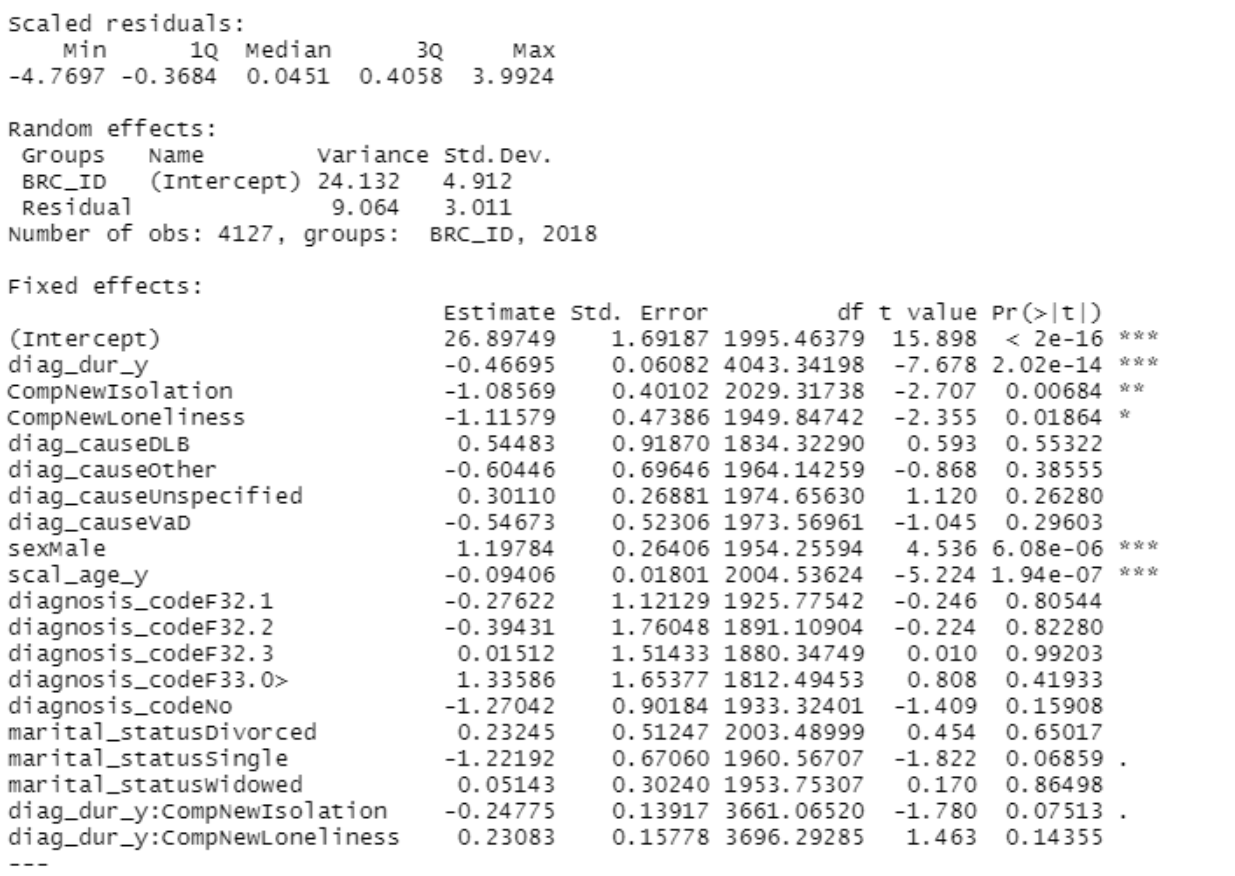


When excluding all patients with missing observation for marital status, we similarly lose more than 50% of patients and their MoCA scores.

The trends again are replicated, with the main effect staying significant while interaction between social isolation and disease duration attenuated.

**
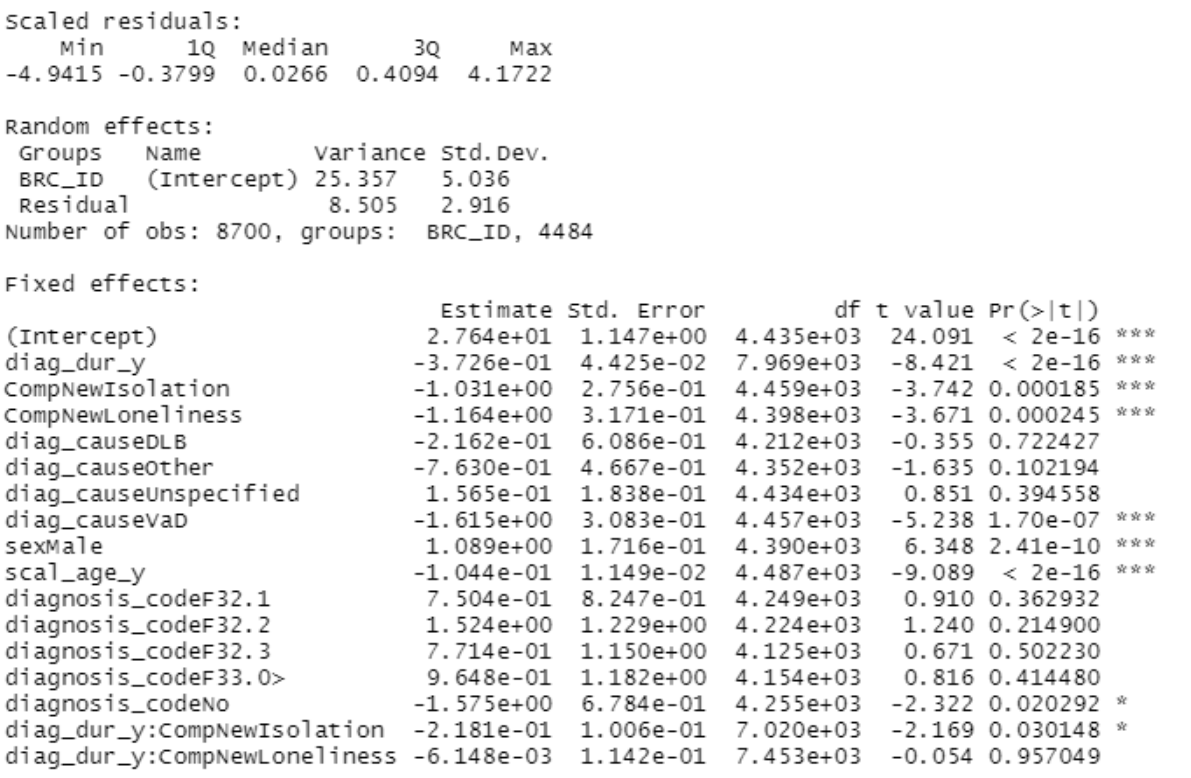
**

**Timing of the social isolation and loneliness report:**

We performed a detailed sensitivity analysis exploring the subset of patients with recorded loneliness or social isolation at varying temporal distances from their dementia diagnosis. Specifically, we examined patients with documented loneliness/social isolation more than three months before their dementia diagnosis, focusing on their diagnostic history and how these factors may influence cognitive trajectories.

**Timeframes**:

**3-month analysis**: We examined records within the timeframe of 3 months before to 3 months after dementia diagnosis to capture the immediate context surrounding the diagnosis.

This substantially reduces the number of cases that we use to estimate trajectories of patients with social isolation and loneliness.


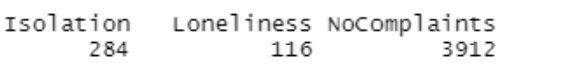


Generalized additive model:


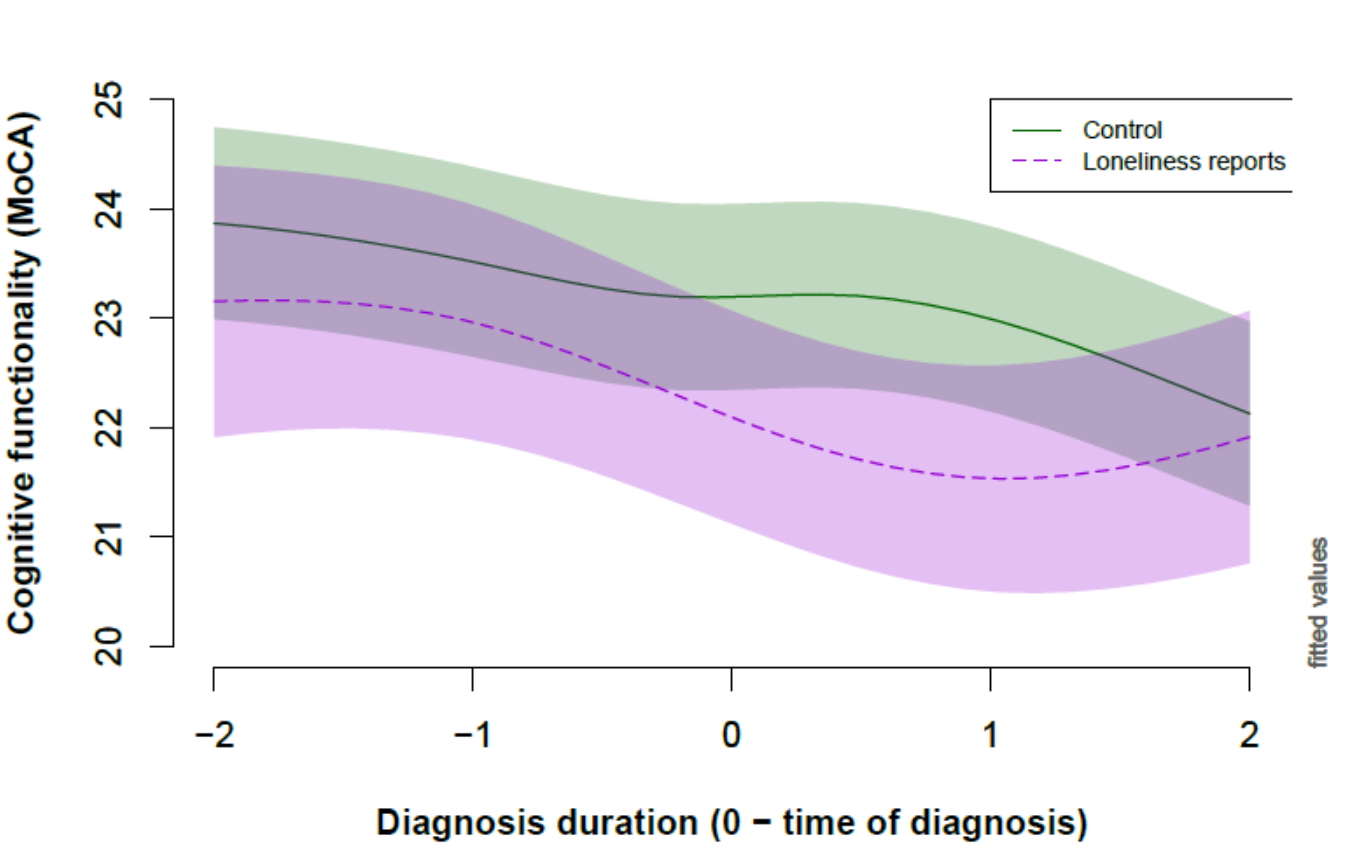


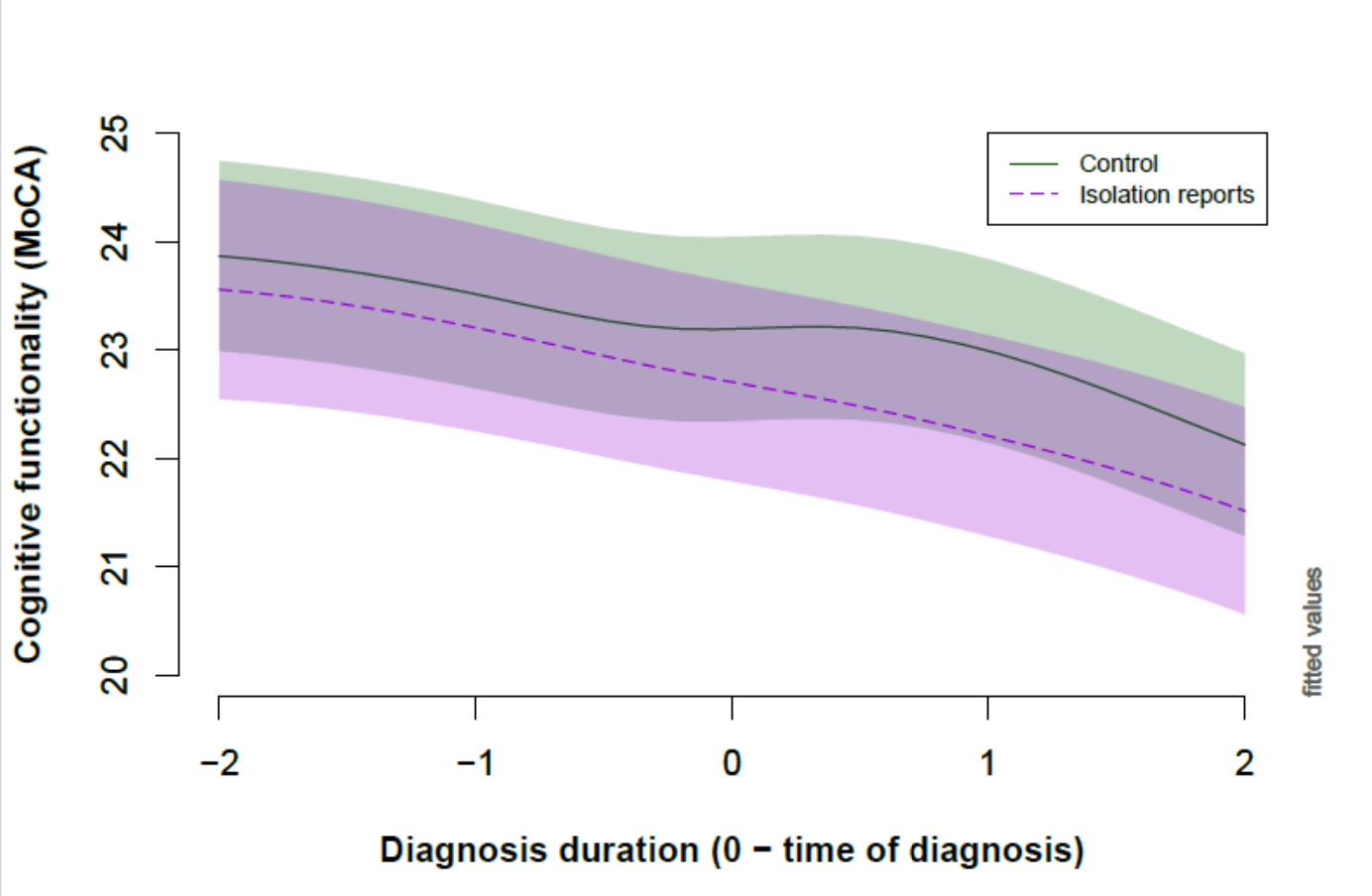


Linear mixed-effect model:


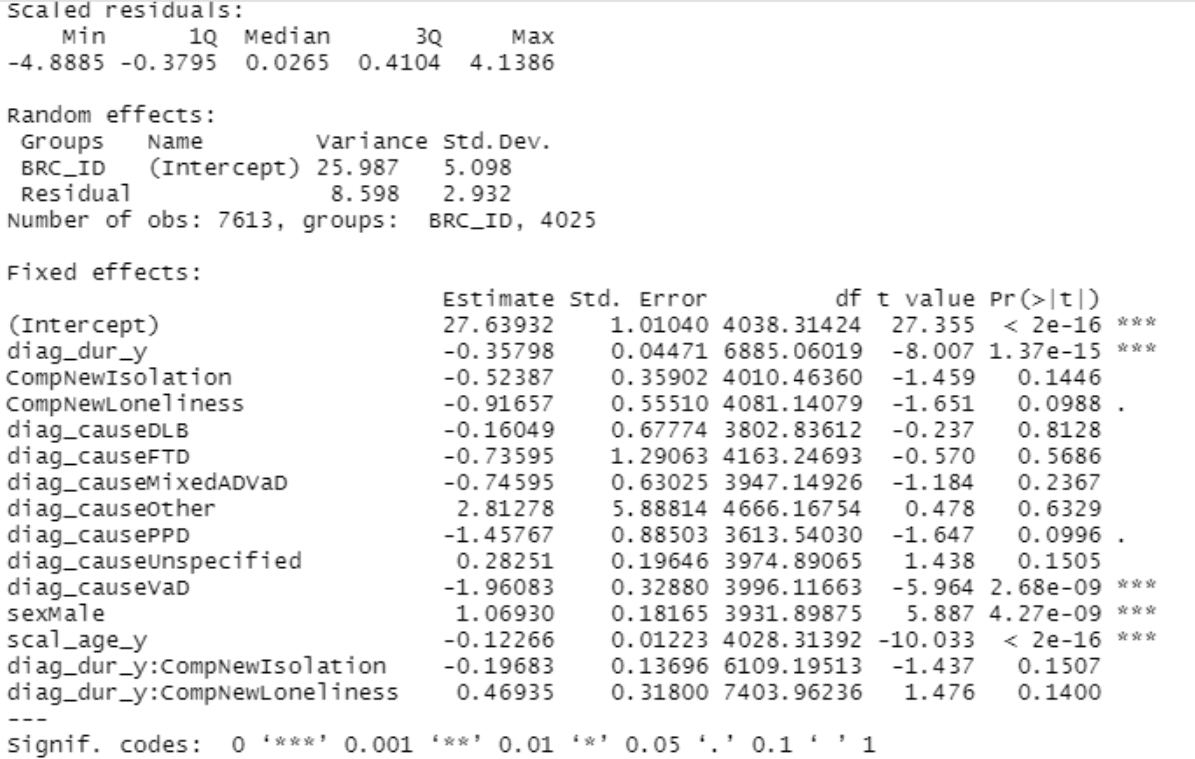


**Extended analysis**: We extended this window to one year before and one year after diagnosis to evaluate broader patterns and ensure robustness of our findings.


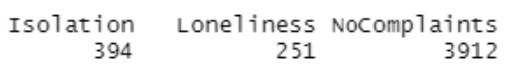


Linear mixed-effect model:


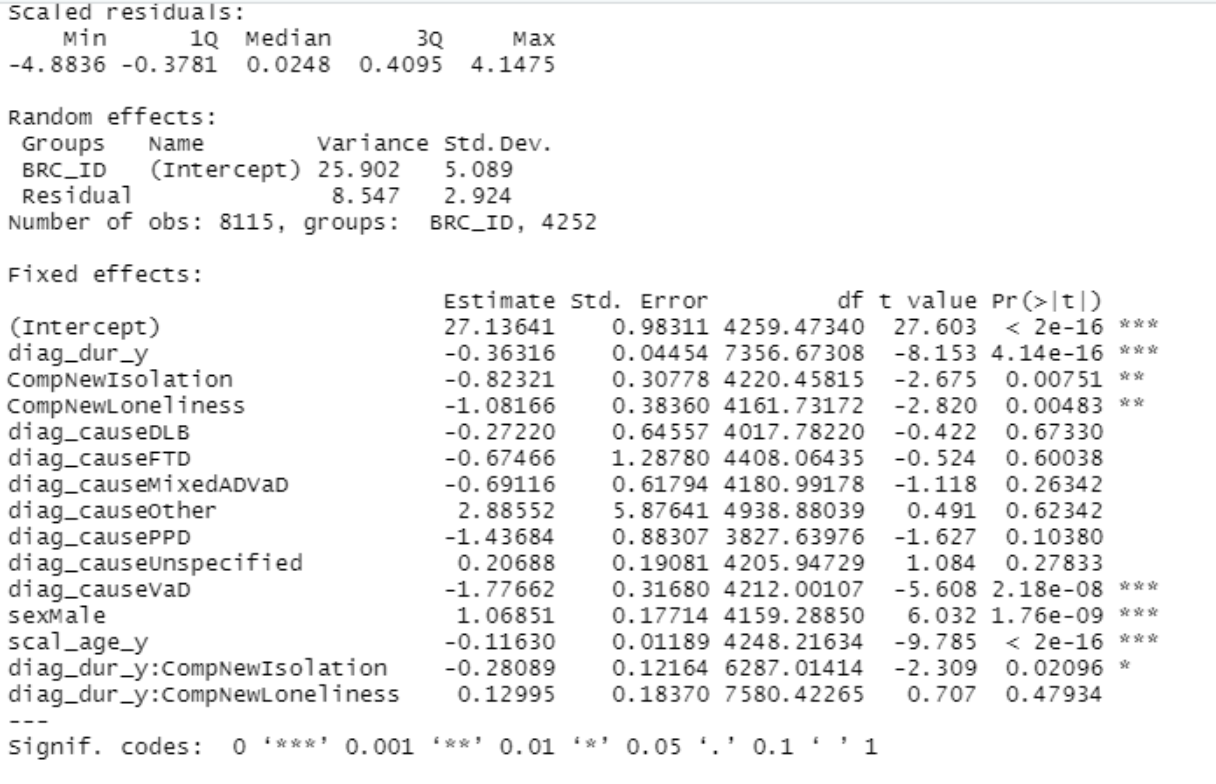


MMSE analysis

We extended our analysis to **MMSE (Mini-Mental State Examination) scores**, following a similar approach used for the MoCA scores.

**GAMM Model**:

We then applied a **generalized additive mixed model (GAMM)**, identical to the model used for MoCA scores, to explore the trajectories of MMSE scores.

This allowed us to compare the **control group** to patients with **social isolation** and **loneliness** reports, incorporating additional **penalizations** on the estimated smooth curves to account for possible overfitting and to regularize the estimates.

**LMER Models**:

Next, we fitted **linear mixed-effect regression (LMER) models** with and without all control variables to assess the effects on MMSE scores.

The LMER models were used to estimate changes in MMSE scores after the first reported instance of social isolation or loneliness, accounting for fixed and random effects.

**Patients with Both Loneliness and Social Isolation**:

For the group of patients reporting **both social isolation and loneliness**, we focused on their **MMSE score trajectories** and compared their rate of cognitive decline to the **control group**.

The results indicated that the **cognitive decline** in the group with both reports was **more pronounced** compared to the control group, suggesting that having both complaints might be associated with a faster decline in MMSE scores.

mmse <- read.csv('MMSECombinedComplaintsClean.csv')

ICD10 <- read_xlsx('F:/22-ExtendedNewMind/New Mind 2/Data_extract_22072024/icd10_depression.xlsx')
ICD10 = ICD10[,c(1,4)]
names(ICD10)[1]='BRC_ID'

ICD10=ICD10[!duplicated(ICD10$BRC_ID),]

mmse<-merge(mmse, ICD10, by='BRC_ID', all.x=T)

mmse2 <- subset(mmse, diag_dur_y >=-5 & diag_dur_y<=5 & (CompNew == 'Isolation' | CompNew == 'Loneliness' | CompNew=='NoComplaints'))

mmse2$marital_status=as.factor(mmse2$marital_status)
levels(mmse2$marital_status)=c('Partnership','Partnership','Divorced','Partnership','Divorced','Single','Widowed')

mmse2$diag_cause=as.factor(mmse2$diag_cause)
levels(mmse2$diag_cause)=c('AD','DLB','Other','Other','Other','Unspecified','VaD')

mmse2$acco_status=as.factor(mmse2$acco_status)
levels(mmse2$acco_status)=c('Other','Mainstream housing','Other','Supported accommodation')

mmse2$diagnosis_code=ifelse(is.na(mmse2$diagnosis_code),'No',mmse2$diagnosis_code)

mmse2$diagnosis_code=as.factor(mmse2$diagnosis_code)
levels(mmse2$diagnosis_code)=c('F32.0','F32.1','F32.2','F32.3','F33.0>','F33.0>','F33.0>','F33.0>','F33.0>','No')

GAMM models

#gam1<-bam(scal_score~CompNew+s(diag_dur_y, by=CompNew, bs='cs')+scal_age_y+diag_cause+sex+s(BRC_ID, bs='re'), data=mmse2)
gamMMSE =readRDS('MMSEIsolationLoneliness.rds')

par(cex=1.1, font.lab=2, font.axis=1.5, bty='n')
plot_smooth(gamMMSE, view='diag_dur_y', rm.ranef = F, se =1, cond=list(CompNew='NoComplaints'), col = c('darkgreen'), xlab='Diagnosis duration (0 - time of diagnosis)', ylab='Cognitive functionality (MMSE)', ylim=c(20,30))

par(new=T)
plot_smooth(gamMMSE, view='diag_dur_y', rm.ranef = F, se =1, cond=list(CompNew='Loneliness'), col = c('darkviolet'), xlab='', ylab='', ylim=c(20,30), rug=FALSE)


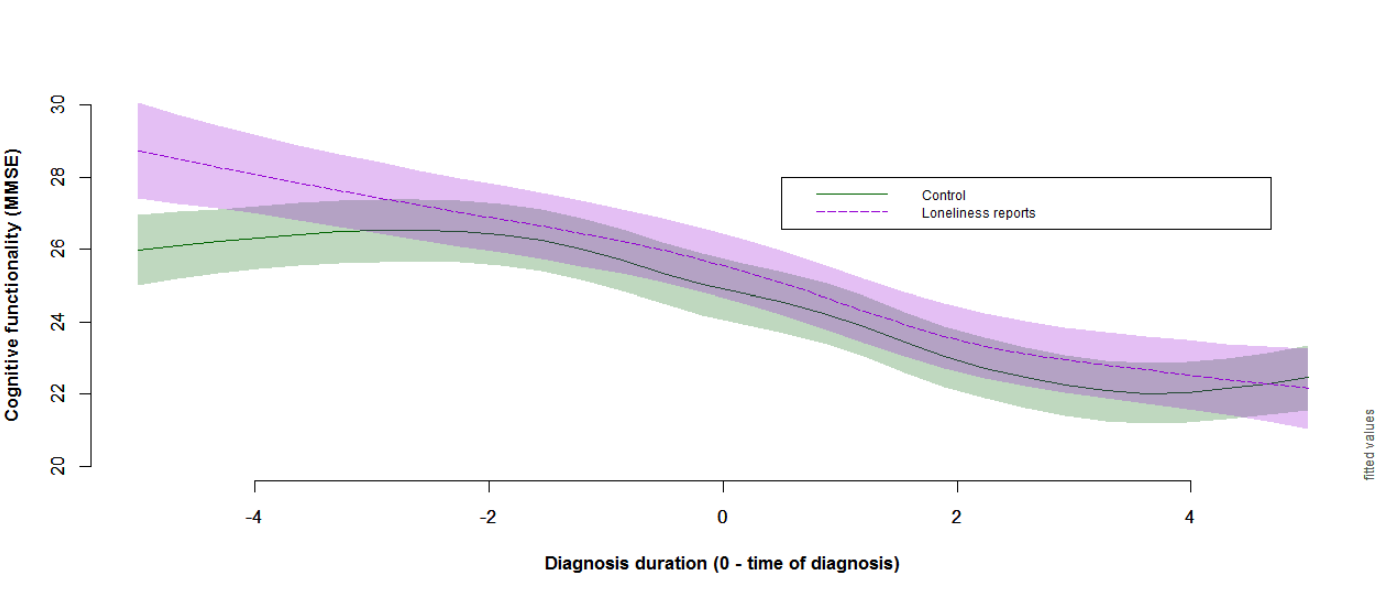


par(cex=1.1, font.lab=2, font.axis=1.5, bty='n')
plot_diff(gamMMSE, view='diag_dur_y', rm.ranef = F, se=1, comp=list(CompNew=c('NoComplaints','Loneliness')),ylab='Estimated difference in MMSE', xlab='Diagnosis duration (0 - time of diagnosis)', main='No complaints minus Loneliness')


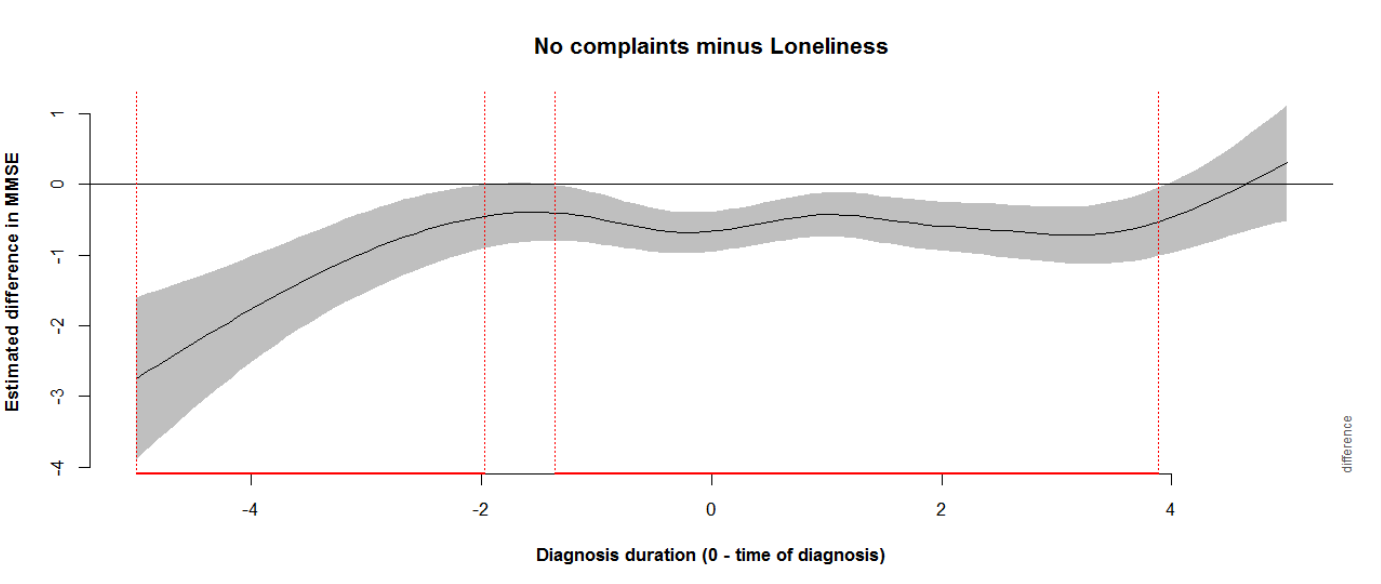
##
## diag_dur_y window(s) of significant difference(s):
## -4.997260 - -1.968618
## -1.362889 - 3.886758

Social isolation:

par(cex=1.1, font.lab=2, font.axis=1.5, bty='n')
plot_smooth(gamMMSE, view='diag_dur_y', rm.ranef = F, se =1, cond=list(CompNew='NoComplaints'), col = c('darkgreen'), xlab='Diagnosis duration (0 - time of diagnosis)', ylab='Cognitive functionality (MMSE)', ylim=c(20,30))

par(new=T)
plot_smooth(gamMMSE, view='diag_dur_y', rm.ranef = F, se =1, cond=list(CompNew='Isolation'), col = c('darkviolet'), xlab='', ylab='', ylim=c(20,30), rug=FALSE)


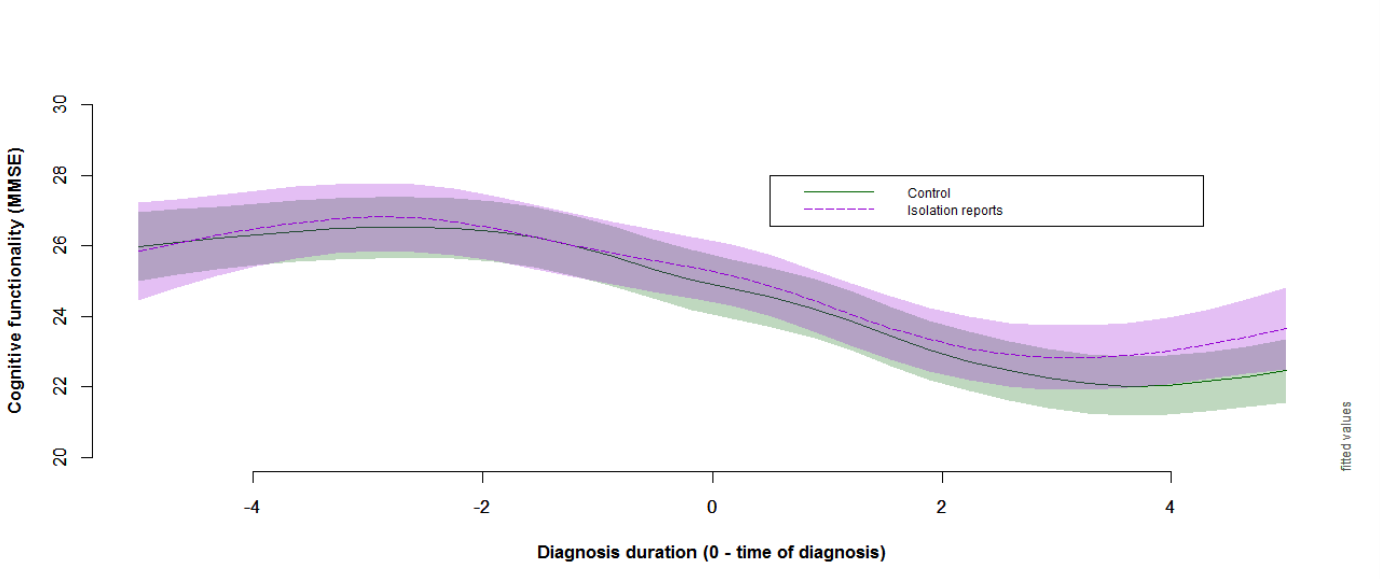


par(cex=1.1, font.lab=2, font.axis=1.5, bty='n')
plot_diff(gamMMSE, view='diag_dur_y', rm.ranef = F, se=1, comp=list(CompNew=c('NoComplaints','Isolation')),ylab='Estimated difference in MMSE', xlab='Diagnosis duration (0 - time of diagnosis)', main='No complaints minus Isolation')


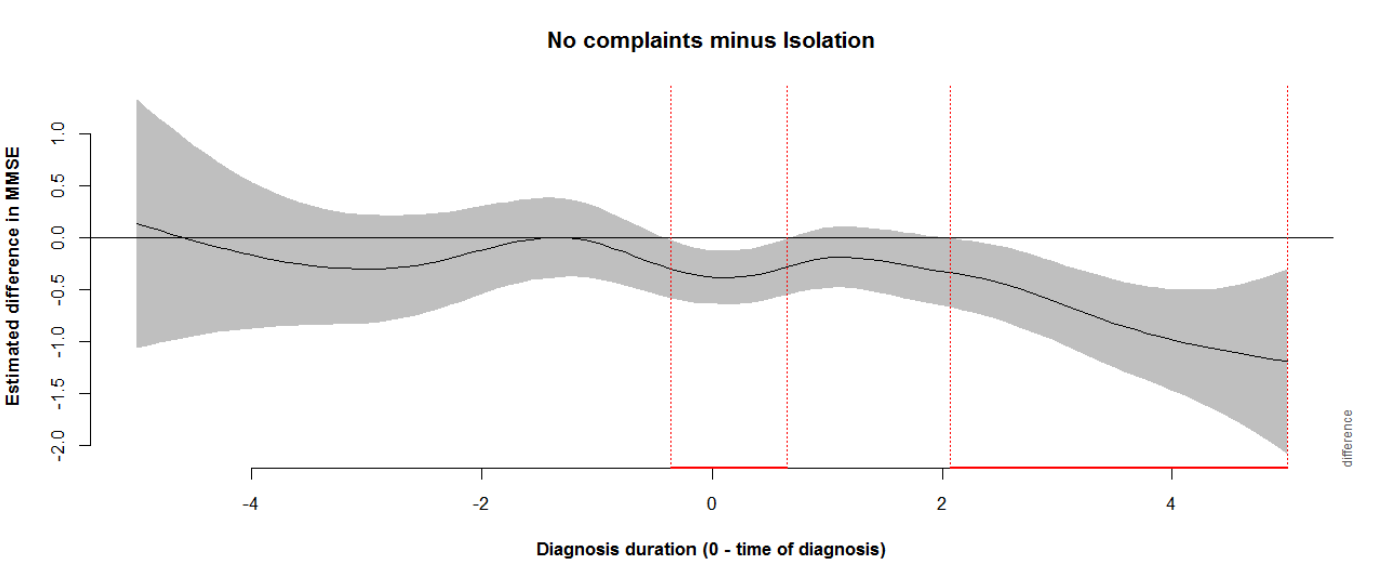


##
## diag_dur_y window(s) of significant difference(s):
## -0.353342 - 0.656206
## 2.069572 - 4.997260

LMER model

Unrestricted and restricted models:

#mmse<-read.csv('MMSECombinedComplaintsClean.csv')
#mmse2=subset(mmse, diag_dur_y >= -5 & diag_dur_y<=5 & CompNew=='Isolation' | CompNew=='Loneliness' | CompNew=='NoComplaints')

mmse2$CompNew=as.factor(mmse2$CompNew)
mmse2$CompNew=relevel(mmse2$CompNew, ref='NoComplaints')

mmse2=mmse2[!is.na(mmse2$diag_dur_y),]

lmerUn1<-lmer(scal_score ~ (1|BRC_ID), data=mmse2)
lmerUn2<-lmer(scal_score ~ diag_dur_y + (1|BRC_ID), data=mmse2)
lmerUn3<-lmer(scal_score ~ diag_dur_y + CompNew + (1|BRC_ID), data= mmse2)
lmerUn4<-lmer(scal_score ~ diag_dur_y * CompNew + (1|BRC_ID), data=mmse2)

anova(lmerUn1, lmerUn2, lmerUn3, lmerUn4)

## refitting model(s) with ML (instead of REML)

## Data: mmse2
## Models:
## lmerUn1: scal_score ~ (1 | BRC_ID)
## lmerUn2: scal_score ~ diag_dur_y + (1 | BRC_ID)
## lmerUn3: scal_score ~ diag_dur_y + CompNew + (1 | BRC_ID)
## lmerUn4: scal_score ~ diag_dur_y * CompNew + (1 | BRC_ID)
## npar AIC BIC logLik deviance Chisq Df Pr(>Chisq)
## lmerUn1 3 100602 100625 -50298 100596
## lmerUn2 4 99607 99638 -49799 99599 996.9439 1 < 2e-16 ***
## lmerUn3 6 99603 99649 -49796 99591 7.8348 2 0.01989 *
## lmerUn4 8 99605 99667 -49794 99589 2.0945 2 0.35091
## ---
## Signif. codes: 0 '***' 0.001 '**' 0.01 '*' 0.05 '.' 0.1 ' ' 1

summary(lmerUn3)

###
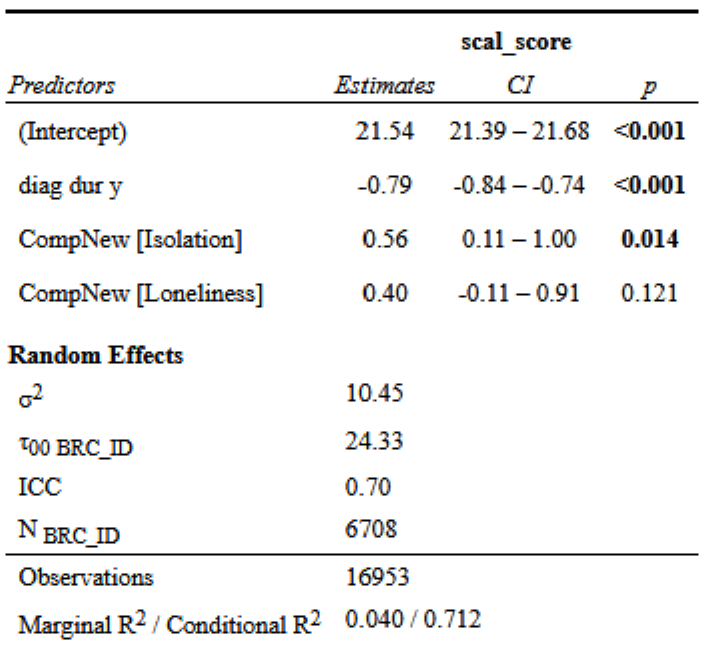


mmse2=mmse2[!is.na(mmse2$sex),]

lmerRe1<-lmer(scal_score ~ diag_dur_y + CompNew + diag_cause + (1|BRC_ID), data=mmse2)
lmerRe2<-lmer(scal_score ~ diag_dur_y + CompNew + diag_cause + sex + (1|BRC_ID), data=mmse2)
lmerRe3<-lmer(scal_score ~ diag_dur_y + CompNew + diag_cause + sex + scal_age_y + (1|BRC_ID), data=mmse2)
lmerRe4<-lmer(scal_score ~ diag_dur_y * CompNew + diag_cause + sex + scal_age_y + (1|BRC_ID), data=mmse2)

anova(lmerRe1, lmerRe2, lmerRe3, lmerRe4)

## refitting model(s) with ML (instead of REML)

## Data: mmse2
## Models:
## lmerRe1: scal_score ~ diag_dur_y + CompNew + diag_cause + (1 | BRC_ID)
## lmerRe2: scal_score ~ diag_dur_y + CompNew + diag_cause + sex + (1 | BRC_ID)
## lmerRe3: scal_score ~ diag_dur_y + CompNew + diag_cause + sex + scal_age_y + (1 | BRC_ID)
## lmerRe4: scal_score ~ diag_dur_y * CompNew + diag_cause + sex + scal_age_y + (1 | BRC_ID)
## npar AIC BIC logLik deviance Chisq Df Pr(>Chisq)
## lmerRe1 10 94134 94211 -47057 94114
## lmerRe2 11 94115 94200 -47047 94093 20.5712 1 5.745e-06 ***
## lmerRe3 12 93968 94060 -46972 93944 149.3669 1 < 2.2e-16 ***
## lmerRe4 14 93969 94077 -46971 93941 2.5211 2 0.2835
## ---
## Signif. codes: 0 '***' 0.001 '**' 0.01 '*' 0.05 '.' 0.1 ' ' 1

summary(lmerRe3)

###
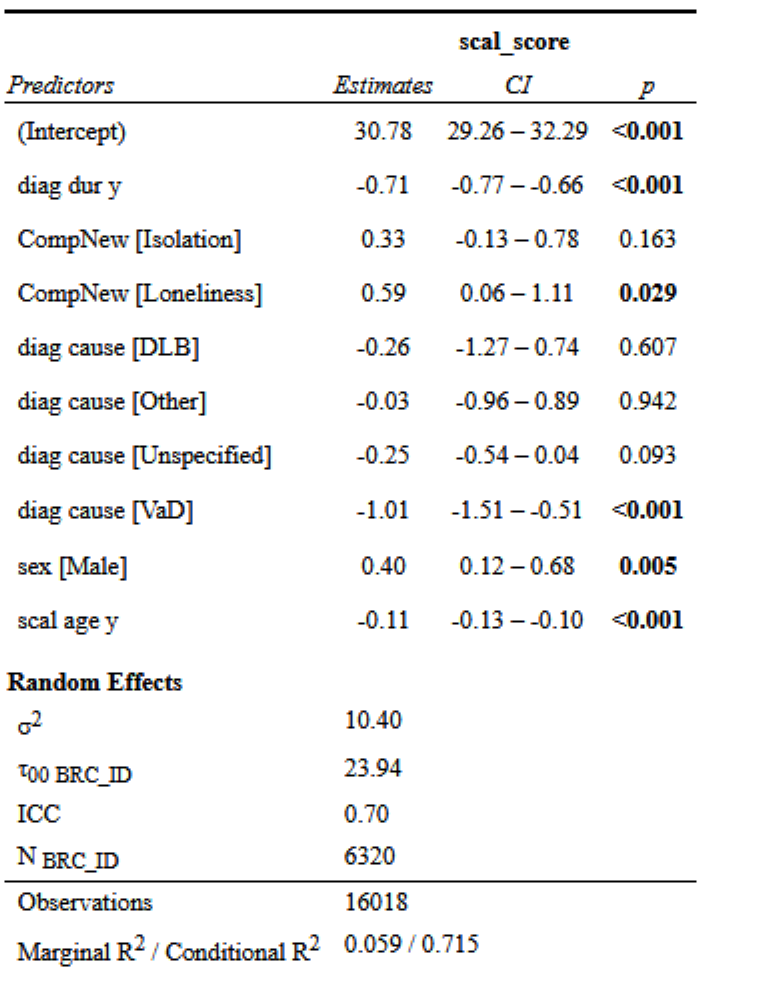


summary(lmerRe4)

###
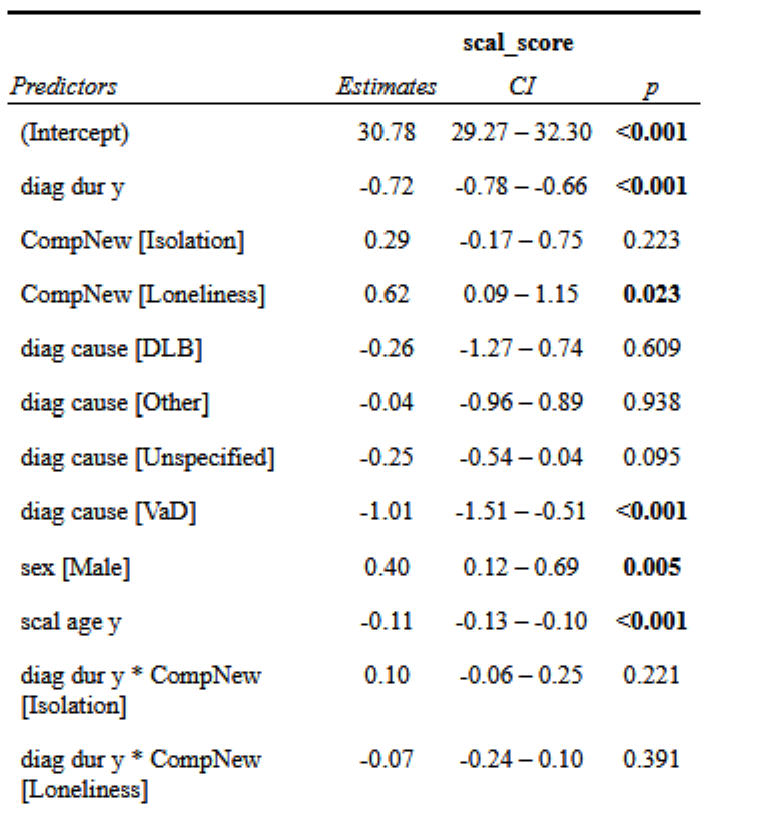


Results from the **LMER models** indicate that patients who reported **social isolation** tend to have **higher MMSE scores** at the point of diagnosis compared to those without such reports. This initial finding suggests that socially isolated patients might have a slightly better cognitive status when diagnosed.

However, when we control for **marital status** and **accommodation status**, this effect becomes **non-significant**. This suggests that the initially observed difference in MMSE scores is largely explained by factors related to whether the patient is married, widowed, or living in supported accommodation. These variables appear to mediate the relationship between social isolation and cognitive performance at diagnosis, rather than social isolation itself being a direct predictor of higher cognitive function.

Patients with reports of social isolation and loneliness

We also conducted a separate analysis focusing on patients who reported both social isolation and loneliness. These patients were excluded from the main analysis due to the fact that they appear to experience a more complex disease trajectory compared to patients who reported either social isolation or loneliness alone.

Key Findings:

In our initial analysis, we examined the number of clinical reports generated for each group of patients throughout their disease progression. The results clearly indicated that patients reporting both social isolation and loneliness had significantly more medical documentation over the course of their illness. Specifically:

- Patients with both social isolation and loneliness reports had on average, 300 clinical reports.
- Patients with only social isolation reports averaged 116 medical reports.
- Patients with only loneliness reports averaged 88 medical reports.

This pattern was also reflected in the mentions of social isolation and loneliness within the clinical records:

- The group with both social isolation and loneliness reports had, on average, 6.6 mentions related to these issues.
- Patients with only social isolation mentions averaged 1.6 mentions.
- Patients with only loneliness mentions averaged 1.5 mentions.

However, these patients also had lowest number of MoCA and MMSE observations in comparison to other three groups, making their trajectories problematic to estimate.

These findings suggest that patients experiencing both social isolation and loneliness may have a more complicated disease course, characterized by a greater need for clinical attention, documentation, and potentially more frequent interactions with healthcare providers. The higher number of medical reports and mentions could indicate that these patients are dealing with more severe symptoms, a broader range of comorbidities, or heightened social and emotional challenges.

obs<-read.csv('LonelinesVerSocialIso.csv')
obs2=aggregate(cbind(obs$LoneC,obs$IsoC), list(obs$brc_id), sum)
colnames(obs2)=c('BRC_ID','Loneliness','Isolation')

obs2$Category = ifelse(obs2$Loneliness>0 & obs2$Isolation>0,'both',ifelse(obs2$Loneliness>0 & obs2$Isolation==0,'Loneliness',ifelse(obs2$Loneliness==0 & obs2$Isolation>0,'Isolation','No')))

obs2$reports=obs2$Loneliness+obs2$Isolation

Mentions of social isolation and loneliness in reports:

tapply(obs2$reports, list(obs2$Category), mean)

## both Isolation Loneliness
## 6.637271 1.507619 1.679682

obs3=read.csv('F:\\22-ExtendedNewMind\\PythonEnv\\Clin2022IDs.csv')
t1<-as.data.frame(table(obs3$brc_id))
names(t1)=c('BRC_ID','Freq')
obs4=merge(t1, obs2, by='BRC_ID')

Mean number of medical documents per group:

tapply(obs4$Freq, list(obs4$Category), mean)

## both Isolation Loneliness
## 295.02829 116.29238 88.21641

moca2a = subset(moca, diag_dur_y >= -5 & diag_dur_y<=5)

Number of observations per group:

table(moca2a$CompNew)

##
## both Isolation Loneliness NoComplaints
## 685 1185 851 7262

We further analyzed the group of patients with both social isolation and loneliness complaints using the previously described **generalized additive models (GAMs)** and **linear mixed-effect regression (LMER) models**.

The results from these analyses showed that patients who reported both social isolation and loneliness had **cognitive performance**, as measured by their **MoCA (Montreal Cognitive Assessment) scores**, that was **comparable to the control group** (patients without any reports of social isolation or loneliness).

Throughout the progression of their disease, these patients did not display significant cognitive decline that would distinguish them from the control group in terms of MoCA scores.

This result is somewhat unexpected given the higher number of clinical reports and mentions of social isolation and loneliness in this group. It suggests that while these patients may experience more **complex social and emotional challenges**, their **cognitive abilities** do not necessarily decline at a faster rate compared to those who do not report such issues. These finding highlights that the cognitive trajectory of dementia patients is not solely defined by social factors like isolation and loneliness, and further exploration is needed to understand the interaction between social experiences and cognitive outcomes.

gamAll<-readRDS('AllGroupsGam.rds')

par(cex=1.1, font.lab=2, font.axis=1.6, bty='n')
plot_smooth(gamAll, view='diag_dur_y', rm.ranef = F, se =1, cond=list(CompNew='NoComplaints'), col = c('darkgreen'), xlab='Diagnosis duration (0 - time of diagnosis)', ylab='Cognitive functionality (MoCA)', ylim=c(12,20), rug=FALSE)

par(new=T)
plot_smooth(gamAll, view='diag_dur_y', rm.ranef = F, se =1, cond=list(CompNew='both'), col = c('darkviolet'), xlab='', ylab='', ylim=c(12,20), rug=FALSE, lty=5)

legend(0.5,20, legend=c('Control','Loneliness & Social isolation reports'), col=c('darkgreen','darkviolet'),lty=c(1,5), cex=0.7)


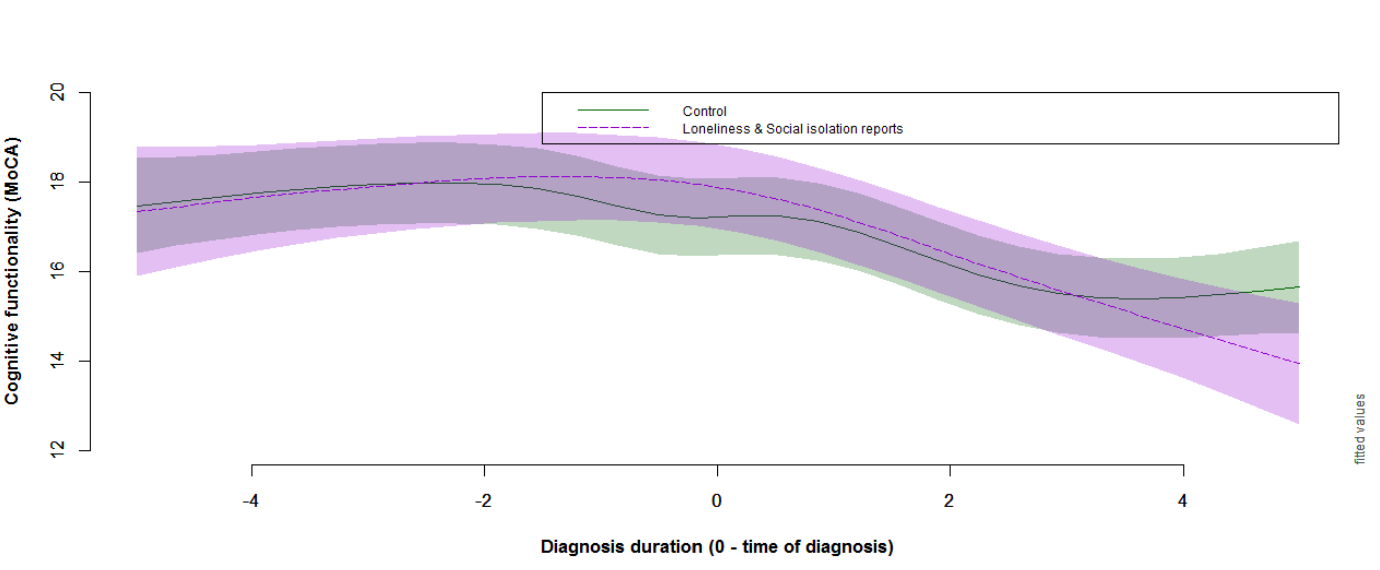


lmerBoth<-lmer(scal_score ~ diag_dur_y * CompNew + diag_cause + sex + scal_age_y + diagnosis_code+marital_status+acco_status+(1|BRC_ID), data=moca2a)

summary(lmerBoth)


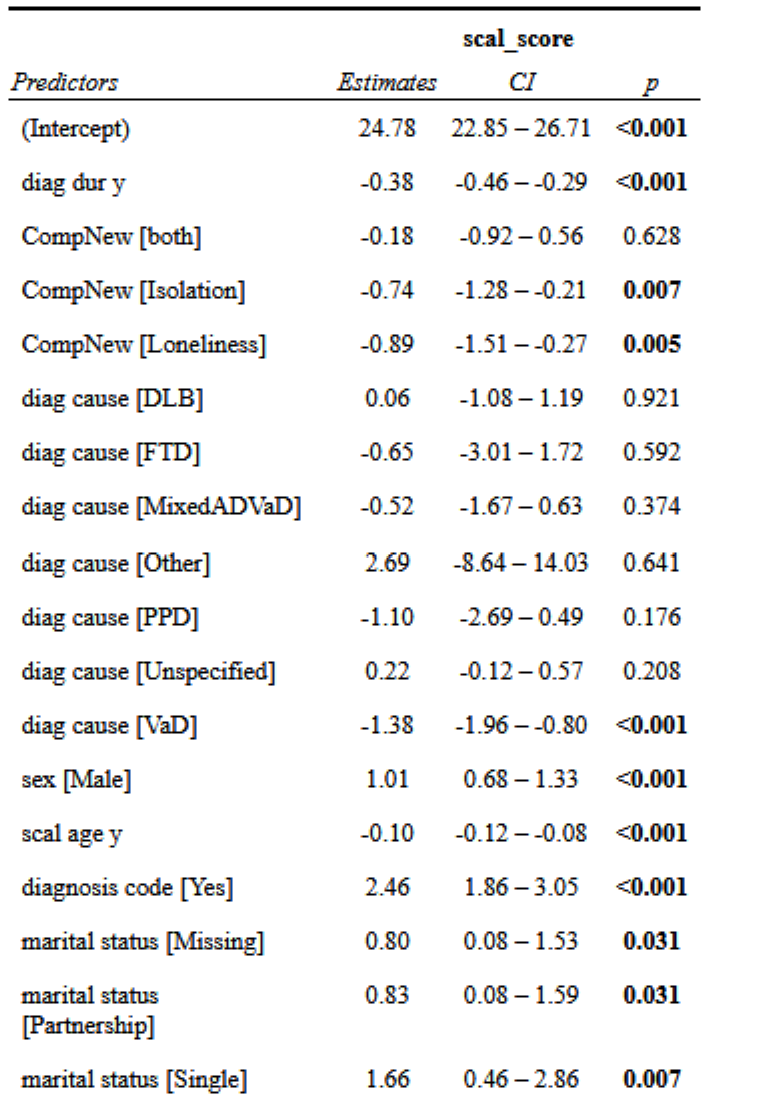


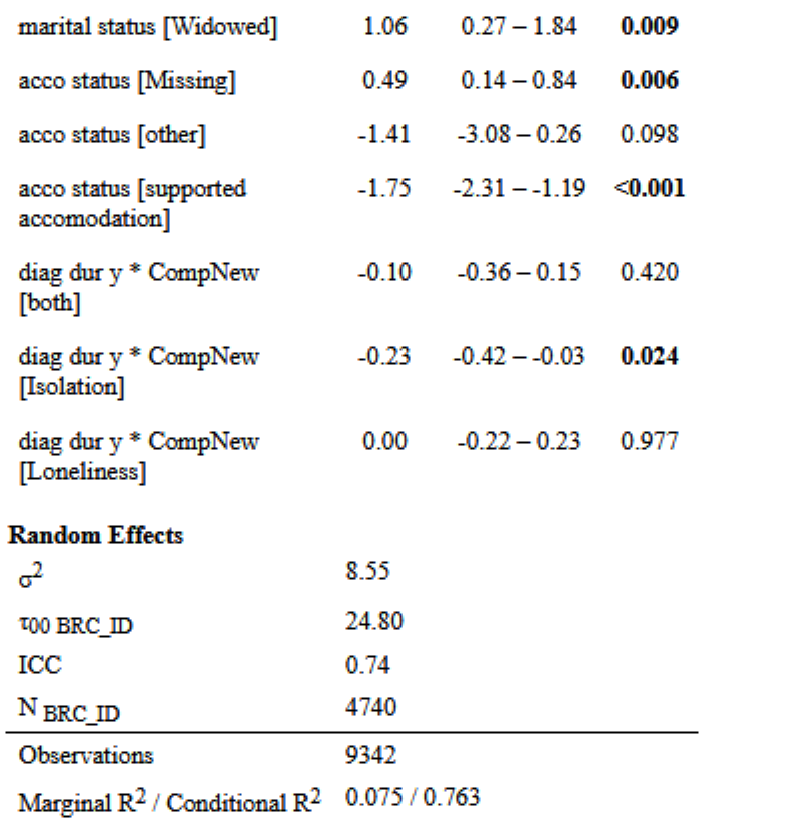

Supplement: Supplementary file 3 — Supporting Information [file DAD2-17-e70149-s001.docx]
